# Diagnostic plasma small extracellular vesicles miRNA signatures for pancreatic cancer using machine learning methods

**DOI:** 10.1016/j.tranon.2023.101847

**Published:** 2023-11-30

**Authors:** Xiaofan Pu, Chaolei Zhang, Guoping Ding, Hongpeng Gu, Yang Lv, Tao Shen, Tianshu Pang, Liping Cao, Shengnan Jia

**Affiliations:** a Department of General Surgery, Sir Run Run Shaw Hospital, School of Medicine, Zhejiang University, Hangzhou, China; b Department of Emergency Medicine, Sir Run Run Shaw Hospital Xiasha Campus, School of Medicine, Zhejiang University, Hangzhou, China; c Department of Thoracic Surgery, Sir Run Run Shaw Hospital, Zhejiang University School of Medicine, Hangzhou, China; d Zhejiang Engineering Research Center of Cognitive Healthcare, Sir Run Run Shaw Hospital, School of Medicine, Zhejiang University, China

**Keywords:** Small extracellular vesicles, miRNA, Pancreatic ductal adenocarcinoma, Diagnosis, Machine learning, Metastasis, Angiogenesis

## Abstract

**Background:**

Identifying biomarkers may lead to easier detection and a better understanding of pathogenesis of pancreatic ductal adenocarcinoma (PDAC).

**Methods:**

Plasma small extracellular vesicles (sEV) from 106 participants, including 20 healthy controls (HC), 12 chronic pancreatitis (CP) patients, 12 benign pancreatic tumour (BPT) patients, and 58 PDAC patients, were profiled for microRNA (miRNA) sequencing. Three machine learning methods were applied to establish and evaluate the diagnostic model.

**Results:**

The plasma sEV miRNA diagnostic signature (d-signature) selected using the three machine learning methods could distinguish PDAC patients from non-PDAC individuals, HC, and benign pancreatic disease (BPD, CP plus BPT) both in training and validation cohort. Combining the d-signature with carbohydrate antigen 19-9 (CA19-9) performed better than with each model alone. Plasma sEV miR-664a-3p was selected by all methods and used to predict PDAC diagnosis with high accuracy combined with CA19-9. Plasma sEV miR-664a-3p was significantly positively associated with the presence of vascular invasion, lower surgery ratio, and poor differentiation. MiR-664a-3p was mainly distributed in the PDAC cancer stroma, including fibers and vessels, and was accompanied by VEGFA expression. Overexpression of miR-664a-3p could promote the epithelial-mesenchymal transition (EMT) and angiogenesis.

**Conclusion:**

In conclusion, our study demonstrated the potential utility of the sEV-miRNA d-signature in the diagnosis of PDAC via machine learning methods. A novel sEV biomarker, miR-664a-3p, was identified for the diagnosis of PDAC. It can also potentially promote angiogenesis and metastasis, provide insight into PDAC pathogenesis, and reveal novel regulators of this disease.

## Highlights

• Potential utility of sEV-miRNA signature in PDAC diagnosis using machine learning methods.
• A novel sEV biomarker, miR-664a-3p, was identified for the diagnosis of PDAC.
• SEV-miR-664a-3p can potentially promote angiogenesis and metastasis.
• SEV-miR-664a-3p provide new insight into PDAC pathogenesis, and reveal novel regulators of this disease.

## Introduction

Pancreatic ductal adenocarcinoma (PDAC), the most common pathological type of pancreatic cancer, is one of the most highly malignant gastrointestinal tumours. Pancreatic cancer has become the third leading cause of cancer-related death in the United States, with a 5-year survival rate of less than 10% [1]. Lacking typical clinical symptoms and effective diagnostic biomarkers, pancreatic cancer is often diagnosed at an advanced stage [2]. This can lead to missed opportunities for patients to receive surgery at an early stage. Carbohydrate antigen 19‐9 (CA19‐9) is used in the diagnosis and treatment evaluation of PDAC cases. However, CA19-9 has been reported to distinguish PDAC from non-PDAC with a limited sensitivity of 0.80 (0.72–0.8) and specificity of 0.75 (0.68–0.80) [3].

Small extracellular vesicles (SEV or Exosomes) contain various biomolecular cargoes, including proteins, nucleic acids, and lipids [4]. The identification of specific sEV cargo has led to biomarkers being discovered and developed, which can be used for cancer diagnosis and prognosis [5]. In recent years, small EV-derived microRNAs (sEV-miRNAs) have attracted attention as a promising blood-based biomarker for cancer detection [6], [7], [8].

Machine learning has improved the ability to discover relevant features in large and high-dimensional data sets collected from sequencing studies [9,10]. This empowers the revelation of sophisticated patterns and correlations, potentially missed by conventional methodologies. Machine learning algorithms, foster an environment of perpetual learning from novel data. This progressively refines and elevates the processes involved in biomarker identification and, consequently, significantly boosts the precision and accuracy of diagnosis [11,12]. From these specific selected features, supervised machine learning methods have been used to develop classifiers for disease diagnosis. Commonly used machine learning methods include the least absolute shrinkage and selection operator (LASSO), support vector machine (SVM) and random forest (RF). Each algorithm possesses unique variable filtering capabilities, and their combined application can effectively address the limitations of individual methods [13,14]. To enhance accuracy, it may be more effective to employ a combination of multiple methods when screening for diagnostic biomarkers. In our findings, we employed three distinct machine learning algorithms: LASSO, SVM and RF.

Multiple reports have shown that miR-664a-3p, which is genomically located in the intron of RAB3 GTPase Activating Non-Catalytic Protein Subunit 2 (RAB3GAP2), is abnormally expressed in a number of cancers, including gastric cancer, cervical cancer, osteosarcoma, breast cancer, and T-cell acute lymphoblastic leukaemia [15], [16], [17], [18], [19]. Another study demonstrated that there were increased levels of miR-664a-3p in EVs in granulomatosis with polyangiitis (GPA), which could be used to discriminate between active GPA and remission [20]. However, it is unclear whether miR-664a-3p expression is altered in pancreatic cancer tissues or plasma sEV. Additionally, its biological functions in PDAC tumorigenesis, progression, and metastasis remains unknown.

In this study, we identified plasma sEV-miRNA biomarkers associated with PDAC using three supervised machine learning feature selection methods. The candidate plasma sEV-miRNAs were used to establish diagnostic models with or without CA19-9. Then, we assessed the performance of these models in a validation cohort. MiR-664a-3p was screened by all three machine learning methods. Next, we further explored the diagnostic performance, correlation with clinicopathological characteristics, and biological functions of miR-664a-3p in PDAC. The findings of this study highlight the clinical and fundamental significance of sEV-miRNAs in PDAC and shed new light on liquid biopsies using sEV-miRNAs using machine learning methods.

## Materials and methods

### Patient enrollment and sample collection

A total of 106 participants were recruited for the training cohort and validation cohort, including patients with PDAC (n=58), healthy controls (HC, n=20), CP patients (n=12) and BPT patients (n=16). All the participants were enrolled from 2020.8 in Sir Run Run Shaw Hospital, school of medicine, Zhejiang university (Hangzhou, China). Characteristics of participants including age, gender, tumor stage, CA19-9 and CEA level were shown in Table 1.

**Table 1:** Characteristics of participants both in training cohort and validation cohort.

Characteristics		Training Set (n=72)				Validation Set (n=34)			
		PDAC	HC	CP	BPT	PDAC	HC	CP	BPT
N		39	14	8	11	19	6	4	5
Age, year	Median	66	55.5	52.5	51	64	62	42	51
	Range	44-82	28-67	31-74	34-78	34-80	29-68	36-53	18-70
Gender	Male	19	5	6	3	11	4	3	3
	Female	20	9	2	8	8	2	1	2
Stage of PDAC patients	Ⅰ	13				3			
	Ⅱ	9				6			
	Ⅲ	9				6			
	Ⅳ	8				4			
CA199(IU/mL)	Median	184	8.76	20.345	15.2	459.4	5.49	13.33	7.39
	Range	0.6-6566	0.6-21.657	4.59-163.8	0.6-66.77	4.01-8934	0.6-19.94	6.82-117	5.82-76.75
CEA(ng/mL)	Median	2.51	1.46	2.85	1.68	4.7	1.99	1.875	1.98
	Range	1-28.92	0.57-3.04	1.63-5.33	0.88-4.96	1.09-19.38	0.8-3.57	5.2-23.19	1.84-3.11

Blood samples were collected from participants with 10 mL EDTA-coated Vacutainer tubes. All the samples were collected before surgery, chemotherapy, radiotherapy, targeted therapy and immunotherapy. Within two hours of blood collection, plasma was separated by centrifugation at 1,300g for 10 min at 4°C. The samples were stored at –80°C.

### Isolation of sEV and sEV RNA

#### Plasma sEV

For each patient, 1.5 mL of plasma was used, and sEV was isolated by affinity-based binding to spin columns using an exoRNeasy Serum/Plasma Kit (Qiagen, Hilden, Germany) following the manufacturer's instructions.

#### Culture medium sEV

For sEV isolation from culture medium, cells were cultured in serum-free medium for 48 h and the culture supernatant was collected. Then the tubes were immediately centrifuged at 3,000 g for 20 min at 4°C to remove cells and debris and filtered on a 0.22 μm filter. Next, the sEV was isolated by using Cell Culture Media Exosome Purification Kits (Norgen Biotech Corp., Thorold, ON, Canada).

#### sEV RNA

RNAs were extracted using a Norgen Exosomal RNA Isolation Kit (Norgen Biotek, 58000, Canada) in accordance with the manufacturer's instructions.

### Small RNA sequencing and bioinformatics analysis

The experimental workflow was performed according to standard procedures provided by Illumina, including library preparation and sequencing experiments. Small RNA sequencing libraries were prepared using TruSeq Small RNA Sample Prep Kits (Illumina, San Diego, USA). After the library preparation was completed, the constructed library was sequenced using Illumina Hiseq2000/2500, and the sequencing read length was single-ended 1 × 50bp. The miRNA data analysis software provided by LC Biology is ACGT101-miR (LC Sciences, Houston, Texas, USA) independently developed by the company. The analysis process is as follows (clean reads are obtained after the raw data is processed by quality control, the 3′ joints are removed from the clean reads, and length screening is performed to keep the base length in 18-25nt (plant) or 18-26nt (animal) sequence. Align the remaining sequences against various RNA database sequences (excluding miRNAs), such as mRNA data library, RFam database (including rRNA, tRNA, snRNA, snoRNA, etc.) and Repbase database (repeated sequence database), and filtered, and finally obtained valid data and it can be used for subsequent small RNA data analysis. Differential expression of miRNAs based on normalized deep-sequencing counts was analyzed by Student t test in this study. The significance threshold was set to be 0.05.

### RNA sequencing analysis

Cells were treated with hsa-miR-664a-3p mimics or Ctrl mimics for 48 h. Then, total RNA was extracted from cells using TRIzol (Invitrogen). Library construction and sequencing were performed by LC Bio Technology CO.,Ltd (Hangzhou, China). The RNA libraries were sequenced on the illumine Novaseq^TM^ 6000 platform by LC Bio Technology CO.,Ltd (Hangzhou, China).

### Cell culture

The human pancreatic cancer-cell lines ASPC-1, BxPC-3, CFPAC-1, HPC-Y5, MiaPaCa-2 and Panc-1 and the normal pancreatic ductal epithelial cell line HPDE6C7 were obtained from the Chinese Academy of Sciences (Shanghai, China). ASPC-1 and BxPC-3 cells were cultured in RPMI 1640 medium (Meilunbio, Dalian, China) containing 10% fetal bovine serum (FBS, CellMax, China). CFPAC-1 cells were maintained in Iscove's Modified Dulbeccos Medium (IMDM, Meilunbio, Dalian, China) with 10% FBS. HPC-Y5, MiaPaCa-2, Panc-1 and HPDE6C7 cells were cultured in DMEM (Meilunbio, Dalian, China) supplemented with 10% FBS.

### Real-time qPCR

Total RNA was extracted with TRIzol reagent (Invitrogen, USA) and isopropanol precipitation methods. The extracted RNA was quantified by measuring the absorbance at 260 nm with a PuXin UV/visible spectrophotometer TU-1810. For miRNA level detection, the hsa-miR-664a-3p RT-qPCR was carried out using a Bulge-Loop miRNA qRT-PCR Starter Kit from Ribo Biotechnology (Guangzhou, China) with the provided miRNA reference gene (u6). Hsa-miR-664a-3p and u6 RT-specific forward and reverse primers and specific bulge-loop miRNA qRT-PCR primers were designed and purchased from Ribo Biotechnology (Guangzhou, China). For mRNA detection, total RNA (1μg) was removed DNA and performed Reverse transcription with Hifair Ⅱ 1st Strand cDNA Synthesis SuperMix for qPCR. All real-time PCR analyses were performed using gene-specific primers and the LightCycler® 480 real-time PCR system (Roche) using SYBR Premix Ex TaqTM (TaKaRa, RR420A). The relative amounts of each mRNA were analyzed using the comparative CT method, as described by Schmittgen and Livak49. All data are expressed as the mean ± SE of n = 3 independent repeats. All statistical analyses were performed using a two-tailed, paired Student's t-test, and P< 0.05 was considered significant. Primer sequences were listed in Supplementary Table 1.

### Western blot

Cells were lysed with RIPA buffer (#FD009, FUDE, China) containing a mixture of protease inhibitor cocktail kit (#P1049, Biyuntian, China). Then the lysates were cleared by centrifugation and the concentrations of proteins were measured by BCA protein assay kit (#P0009, Biyuntian, China). Each total protein sample (4µg) was separated on SDS-PAGE gels and then transferred to PVDF membranes. After incubation of the membranes with primary antibodies at 4 °C overnight, the samples were incubated with the secondary antibodies conjugated with horseradish peroxidase for 1h at room temperature. The Chemiluminescence signals were visualized using a ChemiDoc Touch Imaging System (Bio-Rad). Below is the detailed antibody information (Anti-β-Actin (ABM40032, Abbkine), Anti-Snail (#3879, CST), Anti-Slug (#9585, CST), Anti-E-Cadherin (ab40772, Abcam), Anti-N-Cadherin (ab18203, CST).

### miRNA fluorescence *in situ* hybridization (FISH)

FISH assays were performed using miRNA Fluorescence *in situ* Hybridization Kit (GenePharma) according to the instructions. Oligonucleotide modified probe labeled with cy3 for hsa-miR-664a-3p was provided by GenePharma. First, formalin-fixed paraffin-embedded sections were preheated for 30 min at 60 °C and fresh xylene was used to remove paraffin from the tissue. Then, sections were rehydrated by 10 min incubations in decreasing concentrations of ethanol, and incubated with Proteinase K solution for 15 min at 37 °C and denaturing solution for 8 min at 78 °C. After dehydration, probes were added in the hybridization solution and incubated for 14 h at 37 °C. Sections were washed and counterstained with DAPI (GenePharma). Images were acquired using a fluorescence microscope (Olympus fv3000).

### Cell transfection

The miR-664a-3p inhibitor, miR-664a-3p mimics, Ctrl mimics and Ctrl inhibitor were purchased from Ribobio Co. (Shanghai, China). The mimics, inhibitor, and negative control were used to transfect CFPAC-1 and MiaPaCa-2 cells with ribo FECTTM CP reagent (Ribobio, Shanghai, China) according to the manufacturer's protocol. Following transfection at 48 h, subsequent experimentation was performed.

### Data and statistical analyses

The raw read count of RNA-seq data was converted to TPM values for scale and normalization across all samples. Variates expressed in less than 25% of the entire samples were excluded, and the remaining features were used for subsequent statistical analyses. The plasma sEV miRNA-seq profiles were randomly distributed in training (n=72) and validation cohorts (n=34). The student t test was used to calculate differential expression exmiRs in 58 PDAC patients and 48 non-PDAC individuals. In addition, the false discovery rates (FDRs) of each marker were controlled by Benjamini-Hochberg method.

We applied several machine learning approaches for feature selection, including LASSO regression, random forest (RF) and support vector machines recursive feature elimination (SVM-RFE). LASSO was implemented using R package “glmnet”, with alpha value equal to 1. Random Forest was implemented using R package “randomForest”. SVM-RFE was implemented using R package “e1071”, "kernlab" and "caret".

LASSO. Least absolute shrinkage and selection operator (LASSO) on binomial logistic regression using the glmnet package in R (v4.0) was used to select relevant miRNAs, by eliminating parameters with a coefficient of 0. One of the advantages to using a LASSO method is that coefficients are shrunk and removed, reducing variance without substantially increasing the bias [21]. The lasso approach permits learning and weighting features most relevant to predicting PDAC/non-PDAC status through variable selection. Ultimately, by combining the selected features, the lasso logistic regression model was used to assign a probabilistic score to all samples that were being analysed both in training cohort and validation cohort.

Random forest. The random forest algorithm (RF) is an ensemble learning method based on bagging, which can handle classification problems well. It introduces randomness, is not easy to overfit, and can handle high-latitude data and big data, with high accuracy [22]. After feature selection, Matlab 2019b was used to develop binary classification model.

SVM-RFE. Support vector machines (SVM) are a powerful tool to analyze data with a number of predictors approximately equal or larger than the number of observations. However, originally, application of SVM to analyze biomedical data was limited because SVM was not designed to evaluate importance of predictor variables [23]. Support vector machine recursive feature elimination (SVM-RFE), can filter relevant features and remove relatively insignificant feature variables in order to achieve higher classification performance [24]. On the basis of using SVM-RFE for screening features, the genetic algorithm (GA) is used to find the best g and c values, and then the GA-SVM algorithm is used for classification.

To compare classifiers, we assessed sensitivity, specificity, positive predictive value (PPV), negative predictive value (NPV), accuracy and the area under the curve (AUC) among the classifiers both in training cohort and validation cohort.

Statistical analyses were performed using GraphPad Prism 9 software (GraphPad Software, La Jolla, CA, USA) to assess differences among experimental groups. Statistical differences were analyzed by two-tailed Student's t-test, paired t test, or one-way ANOVA test followed by Tukey's multiple comparisons. The values of *P < 0.05, **P < 0.01, ***P < 0.001, and ****P < 0.0001 are indicative of statistical significance.

**Fig. 1:**
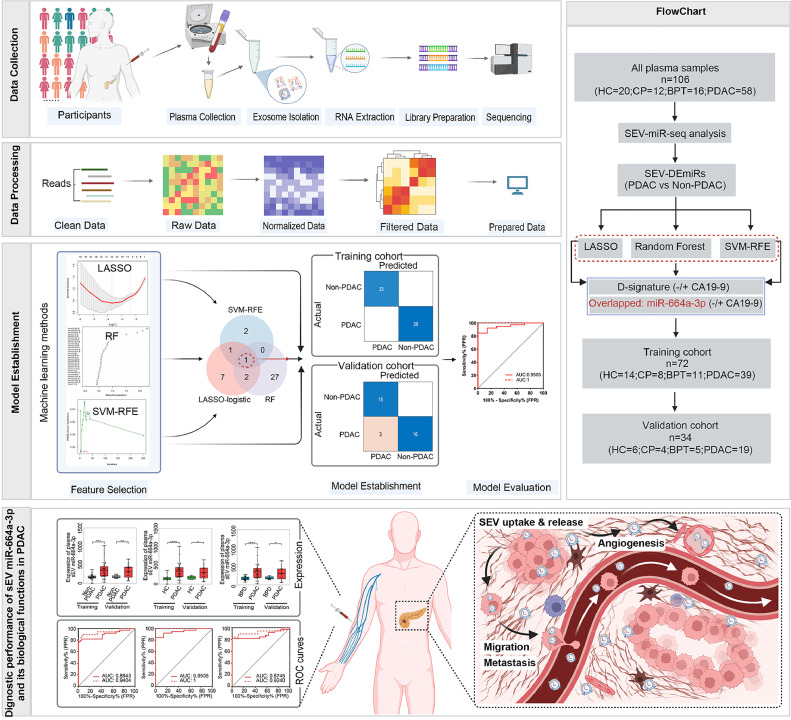
**A summarized diagram of the study and workflow of data generation and analyses.** A total of 106 participants were recruited for the training cohort and validation cohort, including patients with PDAC (n=58), HC (n=20), CP (n=12) and BPT (n=16). MiRNA sequencing was performed after isolation of plasma sEV miRNA. 251 plasma sEV-DEmiRs were differentially expressed in 58 PDAC patients compared with 48 non-PDAC (20HC, 12CP and 16BPT) individuals. To further screen sEV-DEmiRs, three machine learning methods, LASSO regression method, the RF algorithm and SVM-RFE method were used to perform variable selection. The candidate plasma sEV-DEmiRs were used to establish diagnostic models with or without CA19-9 in training cohort. Then, the performance of these models was assessed in validation cohort. MiR-664a-3p was identified by all three feature selection methods. The diagnostic performance of plasma sEV miR-664a-3p in PDAC combined with or without CA19-9 was evaluated both in training cohort and validation cohort. Then the biological functions of sEV miR-664a-3p in PDAC was explored both *in vivo* and *in vitro*. PDAC, pancreatic ductal adenocarcinoma; HC, healthy control; CP, chronic pancreatitis; BPT, benign pancreatic tumor; MiRNA, microRNA; SEV, small extracellular vesicles; DE-miRs, differentially expressed microRNAs; LASSO, least absolute shrinkage and selection operator; RF, random forest; SVM-RFE, support vector machine recursive feature elimination; CA19-9, carbohydrate antigen 19-9.

The workflow was described in Fig. 1.

## Results

### Establishment of sEV-miRNA d-signatures for PDAC using machine learning methods

Overall, 251 plasma sEV-miRNAs were differentially expressed in 58 PDAC patients compared with 48 non-PDAC (20 healthy controls (HC), 12 chronic pancreatitis (CP), and 16 benign pancreatic tumour (BPT)) individuals. To further screen sEV-miRNAs, we used the least absolute shrinkage and selection operator (LASSO) regression method, random forest (RF) algorithm, and support vector machine recursive feature elimination (SVM-RFE) method to perform variable selection.

**Fig. 2:**
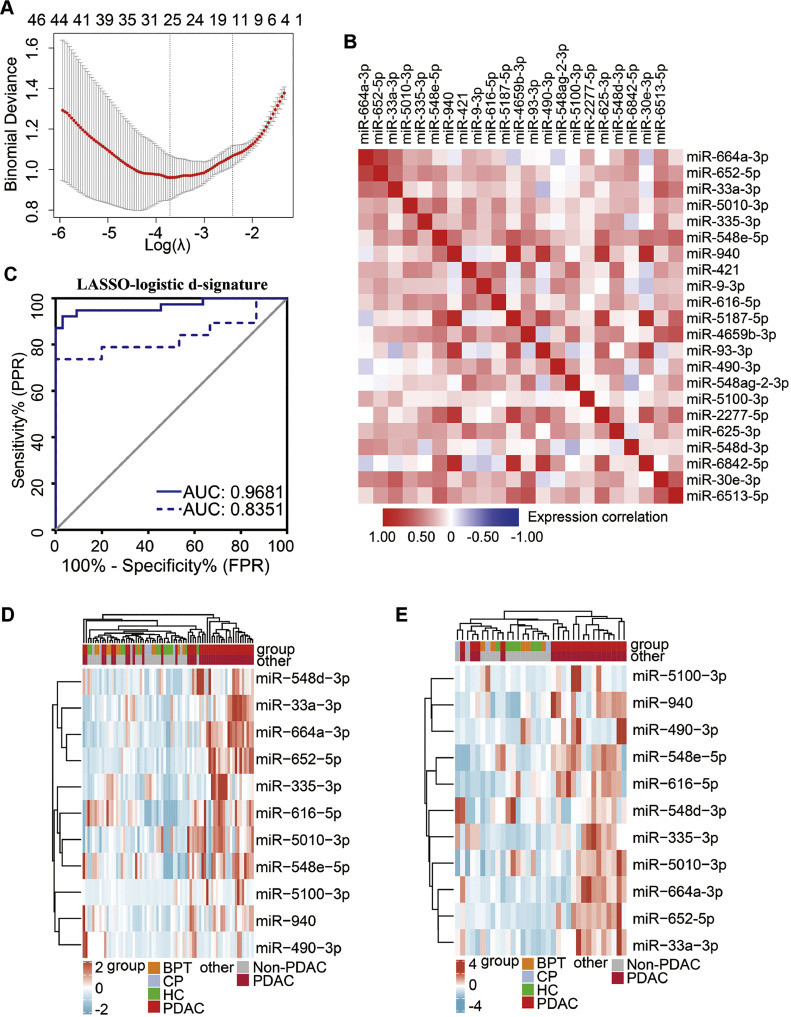
**A plasma sEV miRNAs signature in the diagnosis of PDAC based on LASSO Logistic regression.** (A) The LASSO logistic regression method is used for feature selection), (B) Matrix of correlation (Spearman) between miRNAs selected by LASSO Logistic regression), (C) ROC curves of sEV miRNAs for PDAC patients in training cohort (solid line) and validation cohort (dashed line)), (D) Unsupervised hierarchical clustering of 11 sEV miRNAs selected for use in the d-signature in the training cohort), (E) Unsupervised hierarchical clustering of 11 sEV miRNAs selected for use in the d-signature in the validation cohort. ROC, receiver operating characteristic; AUC, area under the curve; PPR, positive predictive ratio; FPR, false predictive ratio.

Twenty-five sEV-miRNAs were selected using the LASSO regression analysis (Fig. 2A). From these results, we filtered out three sEV-miRNAs (miR-144-5p, miR-1294, and miR-148a-3p) that were downregulated in PDAC patient plasma samples compared with those in samples from non-PDAC individuals. Before multivariate analysis, correlation analysis (Fig. 2B) and collinearity diagnostics (Supplementary Table 2) were conducted to exclude collinearity between features. Finally, 11 sEV-miRNAs (miR-664a-3p, miR-652-5p, miR-33a-3p, miR-5010-3p, miR-335-3p, miR-548e-5p, miR-940, miR-616-5p, miR-490-3p, miR-5100-3p, and miR-548d-3p) remained and were used for logistic regression modelling. The LASSO-logistic sEV-miRNA diagnostic signature (d-signature) comprised the 11 sEV-miRNAs that distinguished PDAC from non-PDAC with an AUC of 0.968, sensitivity of 92.3%, and specificity of 97.0% in the training cohort and an AUC of 0.835, sensitivity of 73.7%, and specificity of 100.0% in the validation cohort (Fig. 2C). To further evaluate the diagnostic performance of the LASSO-logistic sEV-miRNA d-signature among other groups, we also compared the PDAC group with the HC and benign pancreatic disease (BPD), CP and BPT patients) groups. The results showed that the LASSO-logistic d-signature could distinguish PDAC from HC with an AUC of 0.978, sensitivity of 94.9%, and specificity of 100.0% in the training cohort and an AUC of 0.939, sensitivity of 89.5%, and specificity of 100.0% in the validation cohort. The PDAC patients were also distinguished from BPD patients with an AUC of 1, sensitivity of 100.0%, and specificity of 100.0% in the training cohort and an AUC of 0.789, sensitivity of 63.2%, and specificity of 100.0% in the validation cohort (Table 2.1). Unsupervised hierarchical clustering using the 11 sEV-miRNAs could effectively distinguish PDAC from non-PDAC in both the training and validation cohorts (Fig. 2D and E).

**Table 2:** Performance of d-signature in the PDAC using LASSO-logistic regression, RF and SVM-RFE with/without CA19-9.

Table 2.1 Performance of d-signature in the PDAC using LASSO-logistic regression with/without CA19-9.																											
Contrast			PDAC vs Non-PDAC									PDAC vs HC								PDAC vs BPD (CP+BPT)							
Feature			11				11+CA19-9					11					11+CA19-9			11					11+CA19-9		
Group			Training		Validation		Training		Validation			Training		Validation			Training	Validation		Training		Validation			Training		Validation
Sensitivity (%)			36/39 (92.3)		14/19 (73.7)		39/39 (100)		16/19 (84.2)			37/39(94.9)		17/19(89.5)			39/39 (100)	17/19(89.5)		39/39 (100)		12/19 (63.2)			39/39 (100)		14/19 (73.7)
Specificity (%)			32/33(97.0)		15/15 (100)		33/33 (100)		14/15 (93.3)			14/14(100)		6/6(100)			14/14 (100)	6/6(100)		19/19 (100)		9/9(100)			19/19 (100)		9/9(100)
PPV (%)			36/37(97.3)		14/14(100)		39/39 (100)		16/17 (94.1)			37/37(100)		17/17(100)			39/39 (100)	17/17(100)		39/39 (100)		12/12 (100)			39/39 (100)		14/14 (100)
NPV (%)			32/35(91.5)		15/20(75.0)		33/33 (100)		14/17 (82.4)			14/16(87.5)		6/8(75)			14/14 (100)	6/8(75)		19/19 (100)		9/16 (56.3)			19/19 (100)		9/14(64.3)
Accuracy (%)			68/72(94.4)		29/34(85.3)		72/72 (100)		30/34 (88.2)			51/53(96.2)		23/25(92)			53/53 (100)	23/25(92)		58/58 (100)		21/28 (75)			58/58 (100)		23/28(82.1)
AUC			0.968		0.835		1		0.919			0.978		0.939			1	0.974		1		0.789			1		0.877

Table 2.2 Performance of d-signature in the PDAC using RF with/without CA19-9.																											
Contrast	PDAC vs Non-PDAC											PDAC vs HC								PDAC vs BPD (CP+BPT)							
Feature	30					30+CA19-9						30				30+CA19-9				30					30+CA19-9		
Group	Training			Validation		Training				Validation		Training		Validation		Training			Validation	Training			Validation		Training		Validation
Sensitivity (%)	39/39 (100)			13/19 (68.4)		39/39 (100)				16/19 (84.2)		39/39 (100)		19/19(100)		39/39 (100)			19/19(100)	39/39 (100)			14/19 (73.7)		39/39 (100)		16/19 (84.2)
Specificity (%)	33/33 (100)			14/15 (93.3)		33/33 (100)				15/15 (100)		14/14 (100)		4/6(66.7)		14/14 (100)			5/6(83.3)	19/19 (100)			4/9(44.4)		19/19 (100)		2/9(22.2)
PPV (%)	39/39 (100)			13/14(92.9)		39/39 (100)				16/16 (100)		39/39 (100)		19/21(90.5)		39/39 (100)			19/20(95.0)	39/39 (100)			14/19 (73.7)		39/39 (100)		16/23 (69.6)
NPV (%)	33/33 (100)			14/20(70.0)		33/33 (100)				15/18 (83.3)		14/14 (100)		4/4(100)		14/14 (100)			5/5(100)	19/19 (100)			4/9(44.4)		19/19 (100)		2/5(40)
Accuracy (%)	72/72 (100)			27/34(79.4)		72/72 (100)				31/34 (91.2)		53/53 (100)		23/25(92)		53/53 (100)			24/25(96)	58/58 (100)			18/28 (64.3)		58/58 (100)		18/28(64.3)
AUC	1			0.7357		1				0.919		1		0.9524		1			0.98	1			0.4737		1		0.4783

Table 2.3 Performance of d-signature in the PDAC using SVM-RFE with/without CA19-9.																											
Contrast		PDAC vs Non-PDAC									PDAC vs HC										PDAC vs BPD (CP+BPT)						
Feature		4				4+CA19-9					4				4+CA19-9						4			4+CA19-9			
Group		Training		Validation		Training		Validation			Training		Validation		Training			Validation			Training		Validation	Training		Validation	
Sensitivity (%)		28/39 (71.8)		13/19 (68.4)		33/39 (84.6)		16/19 (84.2)			35/39 (89.7)		19/19(100)		35/39 (89.7)			19/19(100)			34/39 (87.2)		14/19 (73.7)	34/39 (87.2)		15/19 (84.2)	
Specificity (%)		32/33 (97.0)		14/15 (93.3)		33/33 (100)		14/15 (93.3)			11/14 (78.6)		4/6(66.7)		11/14 (78.6)			5/6(83.3)			15/19 (78.9)		7/9(77.8)	18/19 (94.7)		8/9(88.9)	
PPV (%)		28/29 (96.6)		13/14(92.9)		33/33 (100)		16/17 (94.1)			35/38 (92.1)		19/21(90.5)		35/38 (92.1)			19/20(95.0)			34/38 (89.5)		14/16 (87.5)	34/35 (97.1)		15/16 (93.8)	
NPV (%)		32/43 (74.4)		14/20(70.0)		33/39 (84.6)		14/17 (82.4)			11/15 (73.3)		4/4(100)		11/15 (73.3)			5/5(100)			15/20 (75)		7/12(58.3)	18/23 (78.3)		8/12(66.7)	
Accuracy (%)		60/72 (83.3)		27/34(79.4)		66/72 (91.7)		30/34 (88.2)			46/53 (86.8)		23/25(92)		46/53 (86.8)			24/25(96)			49/58 (84.5)		21/28 (75.0)	52/58 (89.7)		23/28(82.1)	
AUC		0.8823		0.7895		0.9549		0.8807			0.9469		0.9561		0.9615			0.9737			0.9082		0.7602	0.8815		0.8772	

**Fig. 3:**
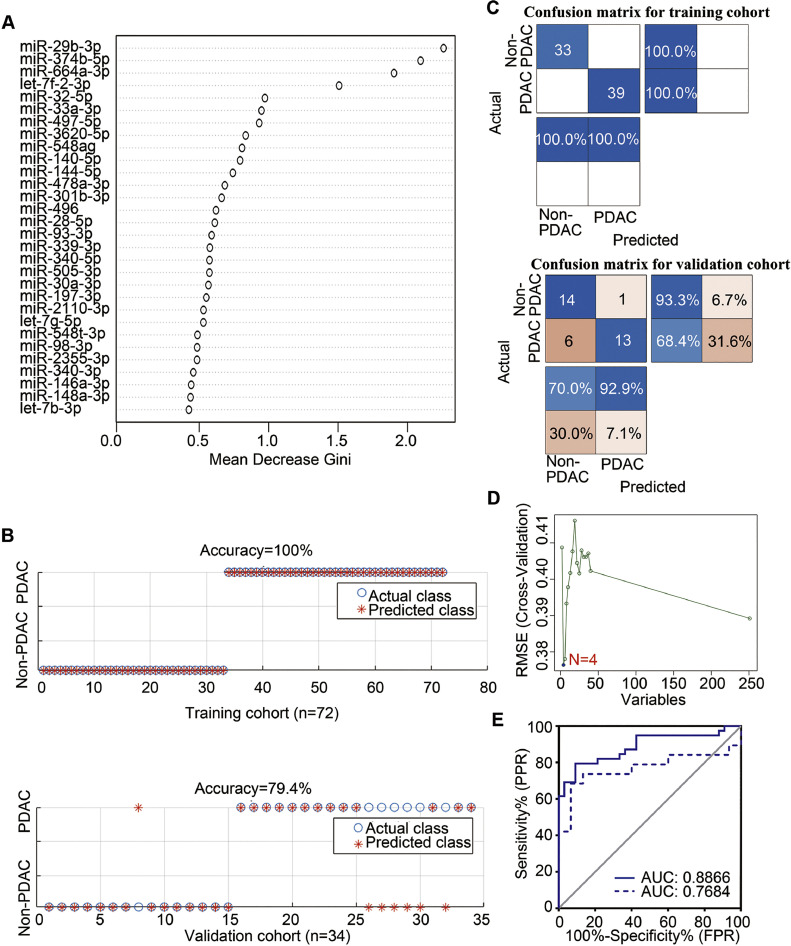
**A plasma sEV miRNAs signature in the diagnosis of PDAC based on random forest machine learning algorithm and SVM-RFE feature selection and SVM classification algorithm.** (A) Variable importance in random forests considering mean decrease in Gini index), (B) Prediction results (training cohort prediction results (upper), validation cohort prediction results (lower)), (C) Confusion matrix of training data (upper) and validation data (lower)), (D) Cross-validated RMSE by recursive feature elimination (RFE) algorithm), (E) ROC curves of 4 sEV miRNAs selected by SVM-RFE for PDAC patients in training set (solid line) and validation set (dashed line). RMSE, root mean square error.

The top 30 sEV-miRNAs (Supplementary Table 3) were selected by Gini importance using the RF algorithm (Fig. 3A). Using the RF method with 30 sEV-miRNAs, we developed a diagnostic model and generated a RF sEV-miRNA d-signature for PDAC. This was able to distinguish PDAC patients from non-PDAC individuals with an accuracy of 100% (Fig. 3B), sensitivity of 100.0%, and specificity of 100.0% in the training cohort (Fig. 3C) and an accuracy of 79.4% (Fig. 3B), sensitivity of 68.4%, and specificity of 93.3% (Fig. 3C) in the validation cohort. When differentiating PDAC patients from HC, the RF sEV-miRNA d-signature displayed an AUC of 1, sensitivity of 100.0%, and specificity of 100.0% in the training cohort and an AUC of 0.952, sensitivity of 89.5%, and specificity of 66.7% in the validation cohort. The RF d-signature appeared to be less effective for distinguishing PDAC from BPD, with an AUC of 1, sensitivity of 100.0%, and specificity of 100.0% in the training cohort and an AUC of 0.470, sensitivity of 73.3%, and specificity of 44.4% in the validation cohort (Table 2.2).

Four sEV-miRNAs (Supplementary Table 4) were identified by using SVM-RFE (Fig. 3D). The GA-SVM was further used to establish a diagnostic model. The results showed that the SVM-RFE d-signature could distinguish PDAC patients from non-PDAC patients with an AUC of 0.882, sensitivity of 71.8%, and specificity of 97.0% in the training cohort and an AUC of 0.79, sensitivity of 68.4%, and specificity of 93.3% in the validation cohort (Fig. 3E). When distinguishing PDAC from HC, for the training set, this model obtained an AUC of 0.947, sensitivity of 89.7%, and specificity of 78.6%, while for the validation set, it had an AUC of 0.956, sensitivity of 100.0%, and specificity of 66.7%. For differentiating PDAC from BPD, the SVM-RFE d-signature had an AUC of 0.908, sensitivity of 87.2%, and specificity of 78.9% in the training cohort and an AUC of 0.760, sensitivity of 73.3%, and specificity of 77.8% in the validation cohort (Table 2.3).

Because CA19-9 has been reported as a blood-based tumour marker for pancreatic cancer [25], in this study, we calculated the AUC of CA19-9 for distinguishing between PDAC patients and non-PDAC individuals. The AUC of CA19-9 was 0.830 (0.732-0.929), with a sensitivity of 71.8% and specificity of 93.9% in the training cohort (Supplementary Fig. 1). This was lower than the AUC values of all three d-signature models. In the validation cohort, the AUC of CA19-9 was 0.8351 (0.700-0.971), with a sensitivity of 57.8% and specificity of 93.3% (Supplementary Fig. 1).

**Fig. 4:**
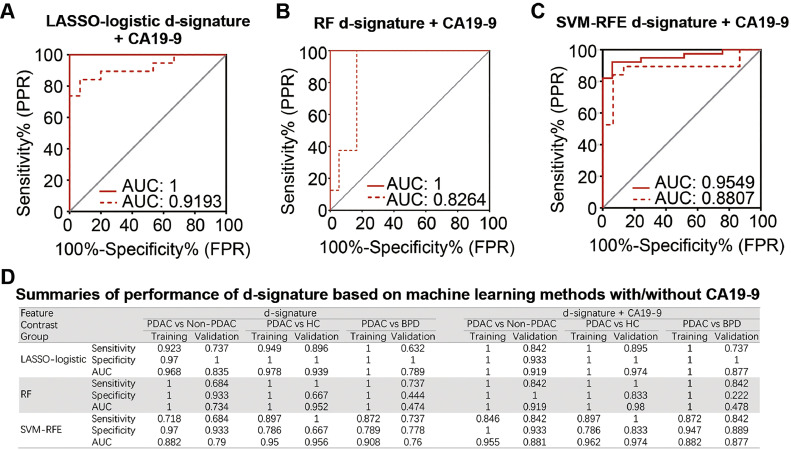
**ROC curves of three plasma sEV miRNAs signatures selected by different machine learning methods combined with CA19-9 for PDAC patients in training cohort (solid line) and validation cohort (dashed line) and summaries of indicators of diagnostic performance.** (A) LASSO logistic regression utilising 11 sEV miRNAs), (B) Random forest algorithm utilising 30 sEV miRNAs), (C) SVM-RFE algorithm utilizing 4 sEV miRNAs. (D) Summaries of performance of d-signature based on machine learning methods with or without CA19-9. D-signature, diagnostic signature.

To further improve the performance of the sEV-miRNA d-signatures, the prediction model was combined with the serum CA19-9. The data suggested that the combination of the sEV-miRNA signature with serum CA19-9 resulted in a better performance than with each model alone. When in combination with CA19-9, the model could distinguish PDAC from non-PDAC and improve the AUC to 1, 1, and 0.955 in the training cohort and to 0.919, 0.919, and 0.881 in the validation cohort by using features selected via LASSO-logistic, RF, and SVM-RFE, respectively (Fig. 4A–C). When differentiating PDAC from HC, features selected by all three machine learning methods combined with CA19-9 had an excellent diagnostic performance with an AUC of 1, 1, and 0.962 in the training cohort and 0.974, 0.962, and 0.974 in the validation cohort via LASSO-logistic, RF, and SVM-RFE, respectively. When distinguishing PDAC from BPD, the LASSO-logistic d-signature and SVM-RFE d-signature combined with CA19-9 had respective AUC values of 1 and 0.908 in the training cohort and 0.877 and 0.877 in the validation cohort. However, the RF d-signature combined with CA19-9 appeared to have a less effective diagnostic performance with an AUC of 1 in the training cohort and 0.478 in the validation cohort (Fig. 4D).

To make a clearer comparison of the performances of the different d-signatures using these three machine learning methods with or without CA19-9, we summarized several indicators in a table (Fig. 4D), including the sensitivity, specificity, and AUC values. More complete and detailed data are presented in Table 2.

### Plasma sEV miR-664a-3p levels can distinguish PDAC from non-PDAC

**Fig. 5:**
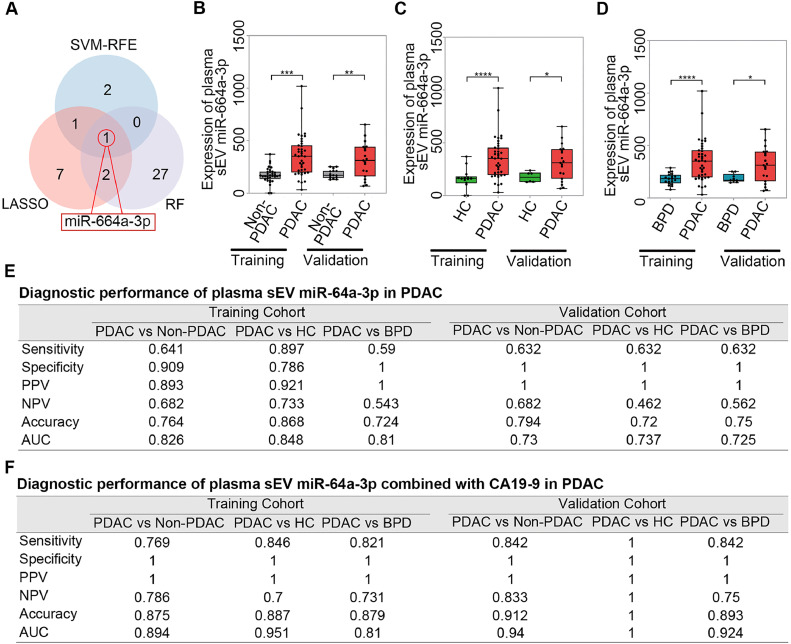
**Plasma sEV miR-664a-3p was identified to distinguish PDAC from non-PDAC with or without CA19-9.** (A) Venn diagram showing overlapping feature selected by LASSO, RF and SVM-RFE), (b–d) The distribution of levels shown as boxplots for the plasma sEV miR-664a-3p from the training cohort and validation cohort in PDAC and other groups (non-PDAC (B), HC (C) and BPD (D)), (E) Diagnostic performance of plasma sEV miR-664a-3p in PDAC), (F) Diagnostic performance of plasma sEV miR-664a-3p in PDAC combined with CA19-9.

Plasma sEV miR-664a-3p was identified by all three feature selection methods (Fig. 5A). To evaluate the potential of sEV miR-664a-3p as a biomarker for PDAC diagnosis, we first compared the expression levels of sEV miR-664a-3p in plasma samples from PDAC patients and non-PDAC individuals. The results showed that plasma sEV miR-664a-3p levels were significantly higher in PDAC patients than in non-PDAC subjects in both the training and validation cohorts (Fig. 5B). In addition, we analysed the expression of plasma sEV miR-664a-3p in HCs and BPD patients. Both the training and validation cohorts showed significant differences in plasma sEV miR-664a-3p levels between PDAC patients and HCs (Fig. 5C) and PDAC patients and BPD patients (Fig. 5D), and was higher in PDAC group. Then, we performed ROC curve analysis to evaluate the diagnostic value of plasma sEV miR-664a-3p in PDAC. Plasma sEV miR-664a-3p levels could be used to distinguish PDAC patients from non-PDAC individuals (AUC=0.826 in the training cohort, AUC=0.730 in the validation cohort), from HC (AUC=0.848 in the training cohort, AUC=0.737 in the validation cohort), and from BPD patients (AUC=0.810 in the training cohort, AUC=0.725 in the validation cohort) (Supplementary Fig. 2A–C). Next, we combined plasma sEV miR-664a-3p with CA19-9 to evaluate its diagnostic performance. The results suggested that the combination led to a greater diagnostic performance for discriminating PDAC from non-PDAC individuals (AUC=0.8943 in the training cohort, AUC=0.940 in the validation cohort), from HCs (AUC=0.951 in the training cohort, AUC=1.000 in the validation cohort), and from BPD patients (AUC=0.810 in the training cohort, AUC= 0.924 in the validation cohort) (Supplementary Fig. 2D–F). More detailed data are summarized in Fig. 5E and F.

To explore miR-664a-3p expression in other tissue types in PDAC patients, such as blood, tumour tissue, and normal-adjacent tissues, we used public databases for further analysis. Serum miR-664a-3p expression levels in PDAC patients and HCs showed no significant difference in the GSE59856 and GSE85589 data sets (Supplementary Fig. 3A and C). ROC curve analysis showed that serum miR-664a-3p can hardly distinguish PDAC patients from healthy individuals in GSE59856 and GSE85589 (Supplementary Fig. 3B and D). There was no differential expression of miR-664a-3p in normal-adjacent and pancreatic cancer tissues by analysing the miRNA-seq data from The Cancer Genome Atlas (TCGA) database in PDAC (Supplementary Fig. 4A). ROC curve analysis showed an AUC of 0.52 (0.19-0.85), indicating a poor diagnostic performance in PDAC patients (Supplementary Fig. 4B). Additionally, Kaplan-Meier survival analysis showed that tissue miR-664a-3p expression levels were not related to the prognosis of PDAC patients (Supplementary Fig. 4C). We further compared the miR-664a-3p expression levels in seven paired cancer and para-cancerous tissues in our centre. A paired Student's t-test was used to assess the statistical significance of this analysis, which suggested that there were no significant differences in miR-664a-3p expression levels between PDAC tissues and their para-cancerous tissues (Supplementary Fig. 4D), *P*=0.8125). To further validate this result, we evaluated miR-664a-3p expression by analysing the GSE119794 cohort from the Gene Expression Omnibus (GEO) database, which contains miRNA-seq profiles of pancreatic cancer in 10 paired tumour and normal pancreatic samples from patients. In accordance with our results, no significant differences were observed between the cancer tissues and para-cancerous tissues (Supplementary Fig. 4E), *P*=0.4316).

Taken together, miR-664a-3p was enriched in plasma sEV of PDAC and was capable of distinguishing PDAC from non-PDAC, HC, and BPD individuals with or without CA19-9. However, tissue expression levels of miR-664a-3p were not significantly different between PDAC and non-cancer tissues.

### MiR-664a-3p is enriched in sEVs and may play a role in PDAC metastasis

**Fig. 6:**
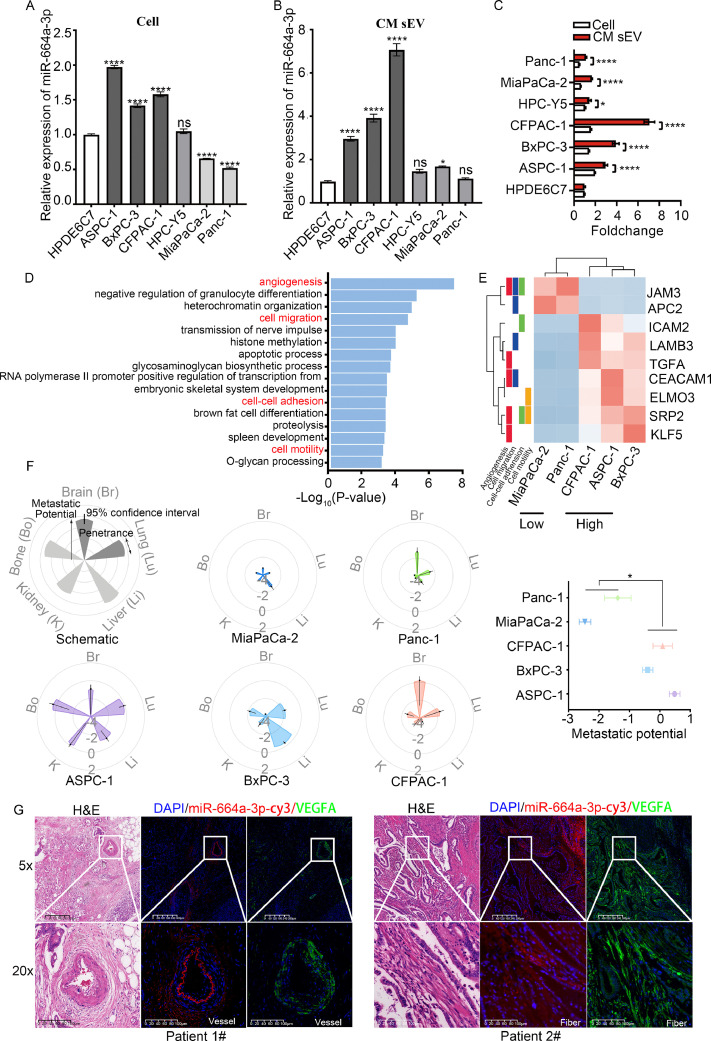
**MiR-664a-3p is enriched in sEV and may play a role in metastasis of PDAC.** (A) Relative expression levels of miR-664a-3p in six PDAC cell lines nomalized by HPDE6C7), (B) Relative expression levels of miR-664a-3p in CM sEV of six PDAC cell lines normalized by HPDE6C7), (C) Expression levels of miR-664a-3p in CM sEV of PDAC cell lines compared to PDAC cell lines normalized by miR-664a-3p in CM sEV of HPDE6C7 and HPDE6C7 cell line), (D) Enrichment of p-value of GO (BP) terms based on DEGs by RNA between high (ASPC-1, BxPC-3, and CFPAC-1) and low (MiaPaCa-2 and Panc-1) miR-664a-3p expression groups), (E) Heatmap of core enrichment genes in angiogenesis, cell migration, cell-cell adhesion and cell motility GO terms in high (ASPC-1, BxPC-3, and CFPAC-1) and low (MiaPaCa-2 and Panc-1) miR-664a-3p expression groups), (F) Schematic of petal plot (first grey one) and five petal plots of different PDAC cell lines (left), forest plot for metastatic potential in five PDAC cell lines (right). Petal length represents metastatic potential. Data are presented on a log10 scale, range from -4 ∼ 4. <= -4: non-metastatic; -4∼-2: (weakly) metastatic, but with low confidence; >= -2: metastatic, with higher confidence. (G) Representative examples of miR-664a-3p *in situ* hybridization, IF staining for VEGFA expression and HE staining on paraffin sections prepared from PDAC patients. CM, culture medium; GO, Gene Ontology; BP, biological process; DEGs, differential expression genes; IF, immunofluorescence; Br, Brain; Lu, Lung; Li, Liver; K, Kidney; Bo, Bone. Error bars represent the means ± SEM of three independent experiments and were analyzed by Student's t-test or ANOVA. *P < 0.05, **P < 0.01, ***P < 0.001, and ****P < 0.0001.

To further investigate the role of miR-664a-3p in PDAC, we measured miR-664a-3p expression levels in six PDAC cell lines and one normal pancreatic ductal epithelial cell line (HPDE6C7) (Fig. 6A). We found that the expression levels of miR-664a-3p in ASPC-1, BxPC-3, and CFPAC-1 cells were relatively higher than in HPDE6C7 cells, while two additional PDAC cell lines, MiaPaCa-2 and Panc-1, expressed lower amounts of miR-664a-3p compared with HPDE6C7. Additionally, there was no significant difference in miR-664a-3p expression between HPC-Y5 and HPDE6C7 cells. These results indicate that miR-664a-3p is expressed at different levels in various PDAC cell lines. Next, we investigated miR-664a-3p expression in sEV extracted from the culture medium (CM) of six PDAC cell lines and HPDE6C7 cells (Fig. 6B). We observed that the expression levels of CM sEV miR-664a-3p were higher in ASPC-1, BxPC-3, CFPAC-1, and MiaPaCa-2 cells than in HPDE6C7 cells. The results also showed that the CM sEV miR-664a-3p levels in HPC-Y5 and Panc-1 cells were not significantly different compared with the levels in HPDE6C7 cells, but the sEV levels were all remarkably higher compared with those in PDAC cell lines normalized to expression in HPDE6C7 (Fig. 6C). The abovementioned data show that miR-664a-3p is enriched in PDAC cell-derived sEV.

After evaluating miR-664a-3p expression levels in PDAC cell lines and their CM sEV, we separated these cell lines into groups with high (ASPC-1, BxPC-3, and CFPAC-1) and low (MiaPaCa-2 and Panc-1) miR-664a-3p expression. To explore potential biological functional differences between the high and low expression groups, we downloaded the microarray expression dataset GSE40098 from the GEO database and analysed 54 differentially expressed genes (DEGs) by using the online analysis tool GEO2R. Biological Process (BP) of gene ontology (GO) analysis revealed 16 significantly enriched GO terms (Fig. 6D). Among them, GO analysis associated these DEGs with vessel formation and adhesion and motility-related pathways, such as angiogenesis, cell migration, cell-cell adhesion, and cell motility (Fig. 6E). These play important roles in the process of metastasis. Additionally, through online analysis and visualization, we compared the metastatic potential of these five PDAC cell lines in MetMap (https://pubs.broadinstitute.org/metmap). The petal plots showed the metastatic ability of the five PDAC cell lines in five organs, including brain, bone, kidney, liver, and lung. Forest plot showed the overall metastatic potential combined with five distant organs of five PDAC cell lines (Fig. 6F). This analysis suggested that ASPC-1, BxPC-3, and CFPAC-1 cells did exhibit higher metastatic abilities than MiaPaCa-2 and Panc-1 cells. Therefore, we hypothesized that miR-664a-3p is associated with PDAC metastasis and is enriched in sEV to exert its functions.

To investigate this further, we analysed the correlation between plasma sEV miR-664a-3p levels and clinicopathological characteristics using the chi‐square test in 58 PDAC patients. The patients were grouped into miR-664a-3p-Low (n=29) or miR-664a-3p-High (n=29) groups based on the miR-664a-3p median expression value. We found that increased plasma sEV miR-664a-3p was significantly positively associated with the presence of vascular invasion (*P*=0.0139) and lower surgery ratio (*P*=0.0178). To some extent, higher levels of plasma sEV miR-664a-3p was positively correlated with advanced TNM stage (*P*=0.0654), the presence of distant metastasis (*P*=0.0656), and poor differentiation (*P*=0.0572), though these results were not statistically significant (Table 3). We also re-analysed the correlation by grouping the patients into the high (upper 75^th^ percentile) or low (lower 75^th^ percentile) expression groups. The results showed that increased miR-664a-3p expression was significantly positively correlated with poor differentiation (*P*=0.0202) and associated with vascular invasion (*P*=0.0588) (Supplementary Table 5). This suggests that higher sEV miR-664a-3p levels indicate a higher degree of malignancy and greater chance of metastasis.

**Table 3:** Plasma sEV miR-664a-3p was correlated with clinical pathologic characteristics of PDAC patients.

Characteristics		Has-miR-664a-3p expression		p value
		High	Low	
Age (years)	≤60	8	11	0.4013
	>60	21	18	
Gender	Male	19	11	0.065
	Female	10	18	
CA199 (U/ml)	<37	7	6	0.7529
	≥37	22	23	
Stage (TNM AJCC 8th)	Ⅰ/Ⅱ	12	19	0.0654
	Ⅲ/Ⅳ	17	10	
Surgery	Yes	11	20	0.0178*
	No	18	9	
Differentiation	Low/middle to low	10	8	0.0572
	Middle/high to middle/high	4	12	
	NA	15	9	
Vascular invasion	Yes	23	14	0.0139*
	No	6	15	
Lymph node metastasis	Positive	13	11	0.5939
	Negtive	16	18	
Distant metastasis	Yes	10	4	0.0656
	No	19	25	

⁎ P < .05; “NA” indicates that no data were available.

Next, we performed fluorescent *in situ* hybridization (FISH) and immunofluorescence staining to detect miR-664a-3p and VEGFA expression in the PDAC tissues. The data showed that miR-664a-3p was mainly distributed in the PDAC cancer stroma, including fibrous tissues and vessels (Fig. 6G), and was positively correlated with VEGFA (Supplementary Fig. 5). This was consistent with former findings in this study, showing that miR-664a-3p may promote angiogenesis and metastasis.

### MiR-664a-3p may promote PDAC metastasis by regulating the epithelial-mesenchymal transition (EMT) and angiogenesis

**Fig. 7:**
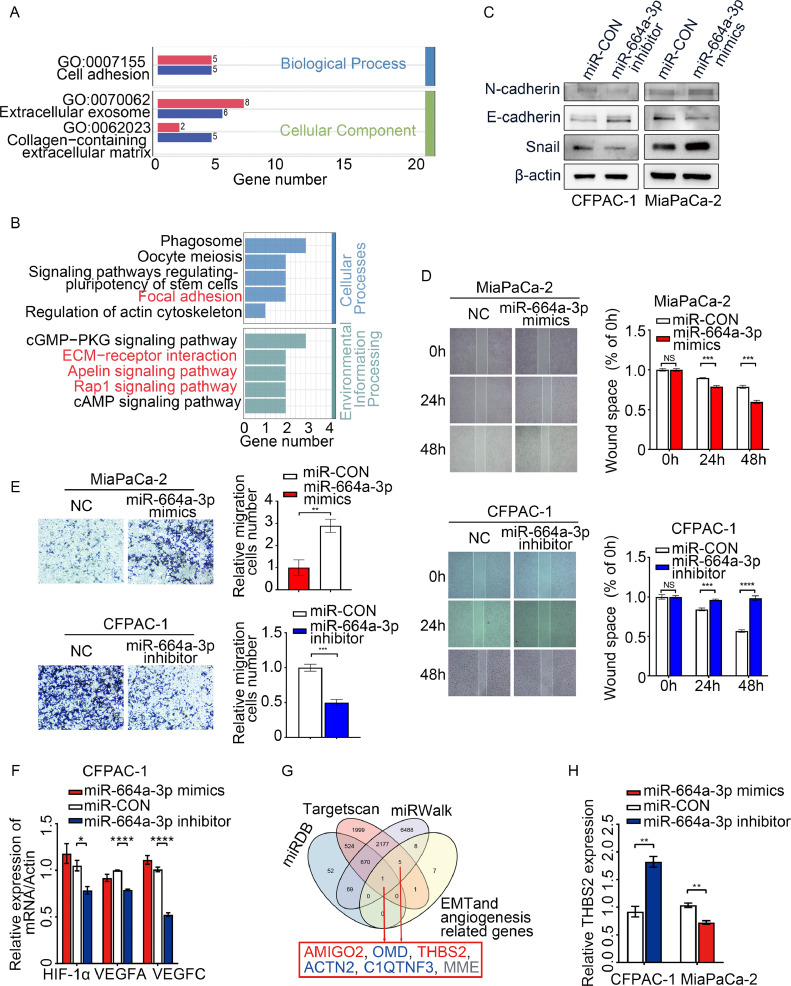
**MiR-664a-3p may promote PDAC metastasis by regulating EMT and angiogenesis.** (A) Enriched GO terms from DEGs by RNA-seq between miR-664a-3p mimics group and NC group in MiaPaCa-2. Representative GO terms (GO:0007155 Cell adhesion, GO:0070062 Extracellular exosome and GO:0062023 Collagen-containing extracellular matrix), (B) Barplot chart for KEGG enrichment analysis from DEGs by RNA-seq between miR-664a-3p mimics group and NC group in MiaPaCa-2.), (C) Western blot analysis for the expressions of E-cadherin, N-cadherin and snail after the treatment with miR-664a-3p inhibitor and mimics, (D) Image of scratch assay and quantification of scratch assay, (E) Image of transwell migration assay and quantification of transwell migration assay, (F) Real-time PCR analyses of representative angiogenic factor mRNAs, HIF-1α, VEGFA and VEGFC in CFPAC-1 treated with miR-664a-3p mimics and inhibitor), (G) Venn diagram of predicted miR-664a-3p targets by four programs (miRDB, Targetscan, miRWalk and pathway related genes concerning EMT and angiogenesis analysed from DEGs)), (H) qRT-PCR analysis of THBS2 in CFPAC-1 upon transfection of miR-664a-3p mimics, inhibitor, and respective controls. DEGs, differential expression genes; KEGG, Kyoto Encyclopedia of Genes and Genomes. Error bars represent the means ± SEM of three independent experiments and were analyzed by Student's t-test or ANOVA. *P < 0.05, **P < 0.01, ***P < 0.001, and ****P < 0.0001.

Then, we investigated the roles of miR-664a-3p in PDAC cell lines. Cell proliferation was not markedly regulated by miR-664a-3p in ASPC-1, BxPC-3, CFPAC-1, MiaPaCa-2, and Panc-1 cells (Supplementary Fig. 6). To gain further insight into the functions of miR-664a-3p in these cell lines, we performed RNA-seq in MiaPaCa-2 cells treated with miR-664a-3p mimics for 48 h. From these results, 114 DEGs were selected for further analysis. The gene ontology (GO) functional terms were identified after functional enrichment analysis of the DEGs (Fig. 7A), and we found enrichment of GO terms associated with cell adhesion, extracellular exosomes, and collagen containing extracellular matrix, which was consistent with effects on cell migration and metastasis. Kyoto Encyclopedia of Genes and Genomes (KEGG) pathway enrichment analysis suggested that overexpression of miR-664a-3p was associated with focal adhesion, extracellular matrix (ECM)-receptor interaction, the Apelin signalling pathway, and the Rap1 signalling pathway (Fig. 7B), which are reportedly related to migration and angiogenesis [26], [27], [28], [29].

Cell adhesion and migration are critical steps in tumour invasion and metastasis. Because the EMT is known to broadly regulate cancer invasion and metastasis [30], we next assessed this in PDAC cells treated with miR-664a-3p mimics or inhibitor by investigating the protein expression levels of EMT markers. Western blot analysis indicated that miR-664a-3p mimic transfection increased the levels of Snail and N-cadherin and attenuated the levels of E-cadherin in MiaPaCa-2 cells. The CFPAC-1 cells treated with the inhibitor showed the opposite trends (Fig. 7C). The scratch assay suggested that miR-664a-3p mimics promoted the migration of MiaPaCa-2 cells, whereas the migration of CFPAC-1 cells were inhibited by miR-664a-3p inhibitor (Fig. 7D). Transwell assays were further performed to investigate the effects of miR-664a-3p on cell migration, and the results were in accordance with those of the scratch assay, indicating decreased migration after miR-664a-3p inhibitor treatment in CFPAC-1 cells and miR-664a-3p mimics treatment in MiaPaCa-2 cells (Fig. 7E). As indicated by the DEGs, angiogenesis was enriched in the miR-664a-3p high expression cell lines compared with that in the low expression cell lines. It was also enriched in the MiaPaCa-2 cells treated with miR-664a-3p mimics compared with that in cells treated with the control mimics. HIF-1α, VEGFA, and VEGFC have been reported to act as factors that contribute to tumour angiogenesis [31]. qRT-PCR was performed to detect the expression levels of these three genes. The results suggested that the HIF-1α, VEGFA, and VEGFC mRNA expression levels were decreased in CFPAC-1 cells treated with the miR-664a-3p inhibitor, while no significant difference was observed when the cells were treated with miR-664a-3p mimics (Fig. 7F). From these data, we can preliminarily conclude that miR-664a-3p can promote EMT and angiogenesis in PDAC cells, thereby increasing the possibility of metastasis. To further explore the target genes of miR-664a-3p, we used online tools (miRDB, Targetscan, and miRWalk) to predict. By overlapping the results of three databases and pathway genes which associated with EMT and angiogenesis, we obtained six genes (AMIGO2, OMD, THBS2, ACTN2, C1QTNF3, and MME) (Fig. 7G). Among these six genes, OMD, ACTN2, and C1QTNF3 were upregulated in miR-664a-3p mimics treated group. And MME level was not detected (value of 0) in miR-664a-3p mimics treated group. Therefore, we selected AMIGO2 and THBS2 as potential targets of miR-664a-3p for validation by qRT-PCR. The result showed that inhibition of miR-664a-3p could significantly augment the expression of THBS2 in CFPAC-1. The cells treated with the mimics showed the opposite trends (Fig. 7H). While the expression level of AMIGO2 was not detected for the possibility of an extremely low abundance. Collectively, THBS2, related to angiogenesis and cancer progression, may be a potential target gene of miR-664a-3p.

## Discussion

In recent years, liquid biopsy has gained increasing attention and been developed as a "non-invasive" diagnostic method. Circulating tumour DNA (ctDNA), circulating tumour cells (CTCs), and EVs have promise for being used as future tools in liquid biopsies. During cell death, ctDNA is formed when fragments of somatic tumour DNA are released into the blood circulation [32]. This can allow for the detection of certain mutations specific to the tumour cells. However, it was reported that in early tumour stages, ctDNA may represent only <1% of total cell-free DNA (cfDNA) [33]. A few studies have even described no detection of ctDNA in early-stage patients, which increases the difficulty of using it as an early diagnostic tool [34]. CTCs can diffuse from the primary tumour site into distant sites through the vasculature. An advantage of CTCs is that they contain RNA, DNA, and protein from the tumour cells, and have a spatial resolution of biomarkers [35]. However, they are poorly enriched in blood, with only 1 to 10 CTCs in 1 mL of blood [36]. This considerably increases the detection difficulty and amount of blood needed. Therefore, the use of sEV can complement and offer an exciting alternative to other liquid biopsy methods for better diagnostic outcomes through the following advantages ((1) they are abundant in the blood and easy to enrich), (2) they are highly stable and easy to preserve), and (3) they can be used to not only diagnose, but also treat diseases.

Recent studies have suggested that sEV miRNAs can serve as non-invasive biomarkers for cancer diagnosis and prognosis [37]. For the first time, Que et al. found that serum-derived sEV miR-17-5p and miR-21 were significantly upregulated in PDAC patients and could be used to distinguish these from HCs and patients with other pancreatic diseases with AUC values of 0.887 and 0.897, respectively [38]. Since then, sEV miR-21 was widely used to distinguish PDAC patients from non-PDAC individuals with an AUC ranging from 0.72 to 1 [39], [40], [41], [42]. In addition to sEV miR-21, several other sEV miRNAs, single or combined, were applied to establish a diagnostic model in PDAC. Lai et al. reported that miR-10b, miR-30c, miR-181a, and miR-let7a were able to distinguish PDAC patients from HCs with an AUC of 1 [8]. Zhou et al. and Xiao et al. also found that sEV miR-10b could serve as biomarker for PDAC diagnosis, with AUC values of 0.88 and 0.99 [41,43]. Those studies, however, were limited by the small number of sEV miRNAs detected in plasma, less varied controls, a lack of a validation set, a relatively small sample size, or a lack of effective calculation methods.

In this study, we obtained miRNA-seq expression profiles from 106 human plasma sEV, including 58 PDAC patients and 48 non-PDAC individuals. Though the sample size was not the largest, we included more comprehensive cases in the non-PDAC group, such as HC, CP patients, and BPT patients. Additionally, we divided the participants into two groups, and along with the training set, a validation set was used to assess the algorithm's effectiveness. To the best of our knowledge, this is the only published plasma sEV miRNA-seq expression profile library that includes all these groups from human plasma sEV.

In addition, we used several machine learning methods to select features, then developed diagnostic models on this basis. In this study, we used three machine learning algorithms to select features. We identified 11, 30, and 4 sEV miRNAs by using the LASSO-logistic regression, RF, and SVM-RFE methods, respectively. After this, we began to establish diagnostic models with or without CA19-9 by using the features selected in the training cohort and re-accessed in a validation cohort. The results suggested that the LASSO d-signature, RF d-signature, and SVM-RFE d-signature could all successfully distinguish PDAC patients from HC with respective AUC values of 0.978, 1, and 0.9469 in the training cohort and of 0.939, 0.9524, and 0.9561 in the validation cohort. When combined with CA19-9, the performances could be improved further, with respective AUC values of 1, 1, and 0.9615 in the training cohort, and of 0.974, 0.98, and 0.9737 in the validation cohort. The LASSO d-signature and RF d-signature performed better than the SVM-RFE d-signature for distinguishing PDAC patients from non-PDAC individuals both in the training and validation sets with or without CA19-9. In addition, the LASSO d-signature performed best for distinguishing PDAC patients from BPD patients with an AUC of 1 in both the training and validation sets. Furthermore, the LASSO d-signature had AUC values of 1 in the training set and 0.877 in the validation cohort when combined with CA19-9. Wang et al. reported in a study that included 17 PDAC and 12 BPD patients that sEV miR-1226-3p had acceptable performance for differentiating PDAC from BPD patients, with an AUC of 0.74 [44].

With machine learning, not only can we predict patient status, but we can also identify previously unknown interactions and identify novel biological features [45]. We then focused on miR-664a-3p because it was selected by all three machine learning methods. Our data are the first to suggest that plasma sEV miR-664a-3p is significantly upregulated in PDAC patients. Plasma sEV miR-664a-3p could distinguish PDAC patients from non-PDAC individuals, HC, and BPD patients with or without CA19-9.

However, there have been no reports regarding sEV miR-664a-3p in PDAC, and reports on miR-664a-3p in other diseases are even more rare. Marcin et al. reported that miR-664a-3p was overexpressed in GPA and could discriminate between active disease and remission [20]. MiR-644a was decreased in blood and tissue samples from Lichen sclerosis (LS) patients compared with that in samples from healthy female volunteers [46]. Apart from the clinical significance of miR-664a-3p, there are several studies that investigated the biological role of this miRNA in cancers and other diseases. He et al. reported that the upregulation of miR-664a-3p inhibits the proliferation of ovarian granulosa cells and increases apoptosis rates by downregulating the expression of BCL2A1 and blocking the MAPK/ERK signalling pathway [47]. However, Wang et al. described that miR-664a-3p was frequently upregulated in GC tissues and cells, and elevated expression of miR-664a-3p could significantly promote cancer cell proliferation and invasion *in vitro* and *in vivo* [15]. These findings suggest that miR-664a-3p plays varied roles in different cases. These studies prompted us to further investigate the role of miR-664a-3p in PDAC, especially sEV miR-664a-3p.

Analysis of miR-664a-3p expression in PDAC cell lines and their CM sEV normalized to the levels in HPDE6C7 showed that miR-664a-3p was enriched in PDAC CM sEV. Similarly, plasma sEV miR-664a-3p was upregulated in PDAC patients, while it showed no significant difference between cancer and para-cancer tissues. These results underscored that miR-664a-3p in sEV plays an important role in pancreatic cancer diagnosis. Further analysis of the correlation between the expression of plasma sEV miR-664a-3p in PDAC patients and clinicopathological characteristics indicated that higher plasma sEV miR-664a-3p levels were positively correlated with the presence of vascular invasion, lower surgery ratio, advanced TNM stage, presence of distant metastasis, and poor differentiation. FISH assay also showed that miR-664a-3p was mainly distributed in neovascularization and fibrous tissues within PDAC stroma. Our study also demonstrated that upregulating miR-664a-3p in CFPAC-1 cells could increase the protein expression levels of EMT markers Snail and N-cadherin and attenuate the levels of E-cadherin. Cells treated with a miR-664a-3p inhibitor showed the opposite expression trends for these markers. Meanwhile, inhibition of miR-664a-3p could significantly decrease the expression of HIF-1α, VEGFA, and VEGFC, which have been reported to act as factors that contribute to tumour angiogenesis. These results suggest that miR-664a-3p is involved in the invasion and metastasis of pancreatic cancer, which are possibly mediated by sEV. Subsequently, we found THBS2 as a downstream target of miR-664a-3p. THBS2, secreted by not only cancer cells but also endothelial cells and immune cells [48], plays an important role in cancer progression, though serving as either a tumour-suppressor or tumour-promoter remains controversial [49]. As a multifunctional glycoprotein, it was involved in angiogenesis, cell motility, and cytoskeletal organization [50,51]. And it has been well acknowledged as potent endogenous inhibitors of angiogenesis [52]. In this study, overexpression of miR-664a-3p could decrease the expression level of THBS2, which may lead to angiogenesis and cancer progression. This brings up a potential target for the treatment of PDAC.

However, there are still several limitations in our study. Firstly, despite the rarity of the sample, the in-house dataset had not enough sample size. Secondly, due to a lack of large and comprehensive public database, we have not performed external validation. Thirdly, though we've demonstrated the diagnostic efficacy of sEV miR-664a-3p in PDAC and conducted initial exploratory direction in angiogenesis and metastasis, the deep mechanism about how sEV transfers miR-664a-3p to recipient cells is not fully elucidated.

In summary, our study used three machine learning feature selection algorithms to identify three diagnostic miRNA signatures for PDAC plasma sEV. These signatures showed robust diagnostic abilities in distinguishing PDAC from HC, non-PDAC patients, and BPD patients. We created an integrated analysis of features selected using the three machine learning methods, then focused on an overlapping miRNA. Plasma sEV miR-664a-3p may serve as a novel PDAC diagnostic biomarker, and higher expression levels are also suggestive of a higher likelihood of tumour invasion and metastasis. Collectively, these sEV miRNAs, especially miR-664a-3p, and their target genes may provide a novel PDAC diagnostic signature, reveal novel disease mechanisms, and highlight future putative drug targets.

## Ethical approval statement

This study was carried out in accordance with The Code of Ethics of the World Medical Association (Declaration of Helsinki) and was approved by the ethics committee of Sir Run Run Shaw Hospital of Zhejiang University (No.20210512-31). Written informed consent was obtained from each participating individuals.

## CRediT authorship contribution statement

**Xiaofan Pu:** Visualization, Methodology, Formal analysis, Writing – original draft, Writing – review & editing. **Chaolei Zhang:** Data curation, Writing – review & editing. **Guoping Ding:** Visualization, Writing – review & editing. **Hongpeng Gu:** Data curation, Writing – review & editing. **Yang Lv:** Formal analysis, Writing – review & editing. **Tao Shen:** Methodology, Writing – review & editing. **Tianshu Pang:** Methodology, Writing – review & editing. **Liping Cao:** Conceptualization, Visualization, Writing – original draft, Writing – review & editing. **Shengnan Jia:** Conceptualization, Visualization, Writing – review & editing.

## Declaration of Competing Interest

The authors declare that they have no known competing financial interests or personal relationships that could have appeared to influence the work reported in this paper.

## Appendix Supplementary materials

Image, application 1

## References

[1] Siegel R.L., Miller K.D., Fuchs H.E., Jemal A. (2022). Cancer statistics, 2022. CA Cancer J. Clin..

[2] Fuchs C.S., Azevedo S., Okusaka T. (2015). A phase 3 randomized, double-blind, placebo-controlled trial of ganitumab or placebo in combination with gemcitabine as first-line therapy for metastatic adenocarcinoma of the pancreas (the GAMMA trial. Ann. Oncol..

[3] Xing H., Wang J., Wang Y. (2018). Diagnostic value of CA 19-9 and carcinoembryonic antigen for pancreatic cancer (a meta-analysis). Gastroenterol. Res. Pract..

[4] Möller A., Lobb R.J. (2020). The evolving translational potential of small extracellular vesicles in cancer. Nat. Rev. Cancer.

[5] Wang M., Ji S., Shao G. (2018). Effect of exosome biomarkers for diagnosis and prognosis of breast cancer patients. Clin. Transl. Oncol..

[6] Xu Y.F., Hannafon B.N., Khatri U., Gin A., Ding W.Q. (2019). The origin of exosomal miR-1246 in human cancer cells. RNA Biol..

[7] Xu Y.F., Hannafon B.N., Zhao Y.D., Postier R.G., Ding W.Q. (2017). Plasma exosome miR-196a and miR-1246 are potential indicators of localized pancreatic cancer. Oncotarget.

[8] Lai X., Wang M., McElyea S.D., Sherman S., House M., Korc M. (2017). A microRNA signature in circulating exosomes is superior to exosomal glypican-1 levels for diagnosing pancreatic cancer. Cancer Lett..

[9] Deo R.C. (2015). Machine learning in medicine. Circulation..

[10] Toh T.S., Dondelinger F., Wang D. (2019). Looking beyond the hype (Applied AI and machine learning in translational medicine. eBioMedicine..

[11] Chi H., Peng G., Wang R. (2022). Cuprotosis programmed-cell-death-related lncRNA signature predicts prognosis and immune landscape in PAAD patients. Cells.

[12] Chi H., Yang J., Peng G. (2023). Circadian rhythm-related genes index: a predictor for HNSCC prognosis, immunotherapy efficacy, and chemosensitivity. Front. Immunol..

[13] Ntakolia C., Kokkotis C., Moustakidis S., Tsaopoulos D. (2021). Identification of most important features based on a fuzzy ensemble technique: evaluation on joint space narrowing progression in knee osteoarthritis patients. Int. J. Med. Inform..

[14] Wadghiri M.Z., Idri A., El Idrissi T., Hakkoum H. (2022). Ensemble blood glucose prediction in diabetes mellitus: a review. Comput. Biol. Med..

[15] Wang L., Li B., Zhang L. (2019). miR-664a-3p functions as an oncogene by targeting Hippo pathway in the development of gastric cancer. Cell Prolif..

[16] Yang Y., Liu H., Wang X. (2015). Up-regulation of microRNA-664 inhibits cell growth and increases cisplatin sensitivity in cervical cancer. Int. J. Clin. Exp. Med..

[17] Bao Y., Chen B., Wu Q. (2017). Overexpression of miR-664 is associated with enhanced osteosarcoma cell migration and invasion ability via targeting SOX7. Clin. Exp. Med..

[18] Wu L., Li Y., Li J., Ma D. (2019). MicroRNA-664 targets insulin receptor substrate 1 to suppress cell proliferation and invasion in breast cancer. Oncol. Res..

[19] Zhu H., Miao M., Ji X. (2015). miR-664 negatively regulates PLP2 and promotes cell proliferation and invasion in T-cell acute lymphoblastic leukaemia. Biochem. Biophys. Res. Commun..

[20] Surmiak M., Wawrzycka-Adamczyk K., Kosałka-Węgiel J., Polański S., Sanak M. (2022). Profile of circulating extracellular vesicles microRNA correlates with the disease activity in granulomatosis with polyangiitis. Clin. Exp. Immunol..

[21] Jiang Y., Wang Y., Zhang J., Xie B., Liao J., Liao W. (2020). Outlier detection and robust variable selection via the penalized weighted LAD-LASSO method. J. Appl. Stat..

[22] Kroell N., Chen X., Maghmoumi A., Koenig M., Feil A., Greiff K. (2021). Sensor-based particle mass prediction of lightweight packaging waste using machine learning algorithms. Waste Manag..

[23] Urwyler P., Rampa L., Stucki R. (2015). Recognition of activities of daily living in healthy subjects using two ad-hoc classifiers. Biomed. Eng. Online.

[24] Huang S., Cai N., Pacheco P.P., Narrandes S., Wang Y., Xu W. (2018). Applications of support vector machine (SVM) learning in cancer genomics. Cancer Genom. Proteom..

[25] Ferrone C.R., Finkelstein D.M., Thayer S.P., Muzikansky A., Fernandez-delCastillo C., Warshaw A.L. (2006). Perioperative CA19-9 levels can predict stage and survival in patients with resectable pancreatic adenocarcinoma. J. Clin. Oncol..

[26] Xie P., Yuan F.Q., Huang M.S. (2021). DCBLD2 affects the development of colorectal cancer via EMT and angiogenesis and modulates 5-FU drug resistance. Front. Cell Dev. Biol..

[27] Vasiukov G., Novitskaya T., Zijlstra A. (2020). Myeloid cell-derived TGFβ signaling regulates ECM deposition in mammary carcinoma via adenosine-dependent mechanisms. Cancer Res..

[28] Uribesalgo I., Hoffmann D., Zhang Y. (2019). Apelin inhibition prevents resistance and metastasis associated with anti-angiogenic therapy. EMBO Mol. Med..

[29] Doebele R.C., Schulze-Hoepfner F.T., Hong J. (2009). A novel interplay between Epac/Rap1 and mitogen-activated protein kinase kinase 5/extracellular signal-regulated kinase 5 (MEK5/ERK5) regulates thrombospondin to control angiogenesis. Blood.

[30] Pastushenko I., Blanpain C. (2019). EMT transition states during tumor progression and metastasis. Trends Cell Biol..

[31] Apte R.S., Chen D.S., Ferrara N. (2019). VEGF in signaling and disease (beyond discovery and development). Cell.

[32] Liang D.H., Hall C., Lucci A. (2020). Circulating tumor cells in breast cancer. Recent Results Cancer Res..

[33] Yong E. (2014). Cancer biomarkers (Written in blood). Nature.

[34] Buscail E., Chauvet A., Quincy P. (2019). CD63-GPC1-positive exosomes coupled with CA19-9 offer good diagnostic potential for resectable pancreatic ductal adenocarcinoma. Transl. Oncol..

[35] Bidard F.C., Peeters D.J., Fehm T. (2014). Clinical validity of circulating tumour cells in patients with metastatic breast cancer (a pooled analysis of individual patient data). Lancet Oncol..

[36] Dawson S.J., Tsui D.W., Murtaza M. (2013). Analysis of circulating tumor DNA to monitor metastatic breast cancer. N. Engl. J. Med..

[37] Reese M., Dhayat S.A. (2021). Small extracellular vesicle non-coding RNAs in pancreatic cancer (molecular mechanisms and clinical implications). J. Hematol. Oncol..

[38] Que R., Ding G., Chen J., Cao L. (2013). Analysis of serum exosomal microRNAs and clinicopathologic features of patients with pancreatic adenocarcinoma. World J. Surg. Oncol..

[39] Pu X., Ding G., Wu M. (2020). Elevated expression of exosomal microRNA-21 as a potential biomarker for the early diagnosis of pancreatic cancer using a tethered cationic lipoplex nanoparticle biochip. Oncol. Lett..

[40] Wu L., Zhou W.B., Zhou J. (2020). Circulating exosomal microRNAs as novel potential detection biomarkers in pancreatic cancer. Oncol. Lett..

[41] Zhou S., Hu T., Han G. (2020). Accurate cancer diagnosis and stage monitoring enabled by comprehensive profiling of different types of exosomal biomarkers (surface proteins and miRNAs). Small.

[42] Goto T., Fujiya M., Konishi H. (2018). An elevated expression of serum exosomal microRNA-191, - 21, -451a of pancreatic neoplasm is considered to be efficient diagnostic marker. BMC Cancer.

[43] Xiao P.P., Wan Q.Q., Liao T., Tu J.Y., Zhang G.J., Sun Z.Y. (2021). Peptide nucleic acid-functionalized nanochannel biosensor for the highly sensitive detection of tumor exosomal MicroRNA. Anal. Chem..

[44] Wang C., Wang J., Cui W. (2021). Serum exosomal miRNA-1226 as potential biomarker of pancreatic ductal adenocarcinoma. Onco Targets Ther..

[45] Lopez-Rincon A., Martinez-Archundia M., Martinez-Ruiz G.U., Schoenhuth A., Tonda A. (2019). Automatic discovery of 100-miRNA signature for cancer classification usingensemble feature selection. BMC Bioinform..

[46] Tan X., Ren S., Yang C. (2021). Differentially regulated miRNAs and their related molecular pathways in lichen sclerosus. Cells.

[47] He M., Mao G., Xiang Y. (2021). MicroRNA-664a-3p inhibits the proliferation of ovarian granulosa cells in polycystic ovary syndrome and promotes apoptosis by targeting BCL2A1. Ann. Transl. Med..

[48] Adolph K.W., Liska D.J., Bornstein P. (1997). Analysis of the promoter and transcription start sites of the human thrombospondin 2 gene (THBS2). Gene.

[49] Nan P., Dong X., Bai X. (2022). Tumor-stroma TGF-β1-THBS2 feedback circuit drives pancreatic ductal adenocarcinoma progression via integrin αvβ3/CD36-mediated activation of the MAPK pathway. Cancer Lett..

[50] Calabro N.E., Kristofik N.J., Kyriakides T.R. (2014). Thrombospondin-2 and extracellular matrix assembly. Biochim. Biophys. Acta.

[51] Jiao H., Zeng L., Zhang J., Yang S., Lou W. (2020). THBS2, a microRNA-744-5p target, modulates MMP9 expression through CUX1 in pancreatic neuroendocrine tumors. Oncol. Lett..

[52] Yang Z., Kyriakides T.R., Bornstein P. (2000). Matricellular proteins as modulators of cell-matrix interactions (adhesive defect in thrombospondin 2-null fibroblasts is a consequence of increased levels of matrix metalloproteinase-2. Mol. Biol. Cell.

