# Supplementary material for: Diagnostic plasma small extracellular vesicles miRNA signatures for pancreatic cancer using machine learning methods
Source: Transl Oncol. 2023 Nov 30;40:101847. doi: 10.1016/j.tranon.2023.101847 (PMC10730862; doi:10.1016/j.tranon.2023.101847)

**Diagnostic plasma small extracellular vesicles miRNA signatures for pancreatic cancer using machine learning methods.**

Xiaofan Pu, Chaolei Zhang, Guoping Ding, Hongpeng Gu, Yang lv, Tao Shen, Tianshu Pang, Liping Cao and Shengnan Jia

**Supplementary materials**

**Table of Contents:**

| Name |  | Heading |
| --- | --- | --- |
| Supplementary Table 1 |  | Primer sequences used for real time PCR. |
| Supplementary Table 2 |  | Collinearity diagnostics performed among sEV-miRNAs selected by LASSO regression. |
| Supplementary Table 3 |  | SEV-miRNAs selected by RF. |
| Supplementary Table 4 |  | SEV-miRNAs selected by SVM-RFE. |
| Supplementary Table 5 |  | Comparison of clinicopathological characteristics of low and high plasma sEV  miR-664a-3p expression groups. |
| Supplementary Figure 1 |  | ROC curves of CA19-9 for PDAC patients and non-PDAC individuals. |
| Supplementary Figure2 |  | ROC curves of sEV miR-664a-3p with/without CA19-9 for PDAC patients and non-PDAC individuals. |
| Supplementary Figure 3 |  | The diagnostic performance of serum miR-664a-3p in PDAC. |
| Supplementary Figure 4 |  | The diagnostic and prognostic performance of tissue miR-664a-3p in PDAC. |
| Supplementary Figure 5 |  | MiR-664a-3p was positively correlated with VEGFA in PDAC stroma. |
| Supplementary Figure 6 |  | MiR-664a-3p have no obvious influence on the proliferation of the PDAC cell lines. |

**Supplementary Table 1: Primer sequences used for real time PCR.**

| Genes |  | Forward primers | Reverse primers |
| --- | --- | --- | --- |
| HIF-1α |  | GAACGTCGAAAAGAAAAGTCTCG | CCTTATCAAGATGCGAACTCACA |
| VEGFA |  | AGGGCAGAATCATCACGAAGT | AGGGTCTCGATTGGATGGCA |
| VEGFC |  | TGTACAAGTGTCAGCTAAGG | CCACATCTATACACACCTCC |
| THBS2 |  | GGGGACACTTTGGACCTCAAC | GCAGCCCACATACAGGCTA |
| AMIGO2 |  | AGAGACTCAGAGGCGACCAT | ATCAGCAAACACAGCAGCTC |
| ACTIN |  | CTCCATCCTGGCCTCGCTGT | GCTGTCACCTTCACCGTTCC |

**Supplementary Table 2: Collinearity diagnostics performed among sEV-miRNAs selected by LASSO regression.**

| MicroRNA | Tolerance | VIF |
| --- | --- | --- |
| hsa-miR-664a-3p | 0.17 | 5.883 |
| hsa-miR-652-5p | 0.253 | 3.959 |
| hsa-miR-33a-3p | 0.259 | 3.867 |
| hsa-miR-5010-3p | 0.347 | 2.884 |
| hsa-miR-335-3p | 0.38 | 2.632 |
| hsa-miR-548e-5p | 0.205 | 4.866 |
| hsa-miR-940 | 0.061 | 16.341 |
| hsa-miR-421 | 0.346 | 2.894 |
| hsa-miR-9-3p | 0.382 | 2.618 |
| hsa-miR-616-5p | 0.514 | 1.945 |
| hsa-miR-5187-5p | 0.068 | 14.807 |
| hsa-miR-4659b-3p | 0.165 | 6.048 |
| hsa-miR-93-3p | 0.115 | 8.694 |
| hsa-miR-490-3p | 0.342 | 2.927 |
| hsa-miR-548ag-2-3p | 0.446 | 2.244 |
| hsa-miR-5100-3p | 0.734 | 1.363 |
| hsa-miR-2277-5p | 0.191 | 5.243 |
| hsa-miR-625-3p | 0.385 | 2.595 |
| hsa-miR-548d-3p | 0.223 | 4.478 |
| hsa-miR-6842-5p | 0.144 | 6.965 |
| hsa-miR-30e-3p | 0.205 | 4.877 |
| hsa-miR-6513-5p | 0.208 | 4.813 |

**Supplementary Table 3: SEV-miRNAs selected by RF.**

| MicroRNA |
| --- |
| hsa-miR-664a-3p |
| hsa-miR-148a-3p |
| hsa-miR-616-5p |
| hsa-miR-33a-3p |
| hsa-miR-140-5p |
| hsa-let-7a-3p |
| hsa-miR-769-3p |
| hsa-miR-589-3p |
| hsa-miR-487a-3p |
| hsa-miR-2110-3p |
| hsa-miR-197-3p |
| hsa-let-7b-3p |
| hsa-miR-505-3p |
| hsa-miR-497-5p |
| hsa-miR-92a-3p |
| hsa-miR-32-5p |
| hsa-miR-29b-3p |
| hsa-miR-98-3p |
| hsa-miR-30a-5p |
| hsa-miR-2355-3p |
| hsa-miR-548t-3p |
| hsa-miR-339-3p |
| hsa-miR-374b-5p |
| hsa-miR-30a-3p |
| hsa-miR-548ag |
| hsa-miR-340-3p |
| hsa-let-7f-2-3p |
| hsa-miR-301b-3p |
| hsa-let-7g-5p |
| hsa-miR-3620-5p |

**Supplementary Table 4: SEV-miRNAs selected by SVM-RFE.**

| MicroRNA |
| --- |
| hsa-miR-664a-3p |
| hsa-miR-652-5p |
| hsa-miR-1185-5p |
| hsa-miR-744-3p |

**Supplementary Table 5: Comparison of clinicopathological characteristics of low and high plasma sEV miR-664a-3p expression groups.** Patients were grouped into the high (upper 75th percentile) and low (lower 75th percentile) levels of miR-664a-3p expression groups.

| Characteristics | | Has-miR-664a-3p expression | | | p value |
| --- | --- | --- | --- | --- | --- |
|  |  | High |  | Low |  |
| Age (years) | ≤60 | 3 |  | 15 | 0.2833 |
|  | ＞60 | 12 |  | 28 |  |
| Gender | Male | 12 |  | 18 | 0.159 |
|  | Female | 3 |  | 25 |  |
| CA199 (IU) | ＜37 | 2 |  | 11 | 0.4796 |
|  | *≥37* | *13* |  | *32* |  |
| Stage (TNM AJCC 8th) | Ⅰ/Ⅱ | 6 |  | 25 | 0.2252 |
|  | Ⅲ/Ⅳ | 9 |  | 18 |  |
| Surgery | Yes | 5 |  | 26 | 0.697 |
|  | No | 10 |  | 17 |  |
| Differentiation | Low/middle to low | 6 |  | 12 | 0.0202* |
|  | Middle/high to middle/high | 0 |  | 16 |  |
|  | NA | 9 |  | 15 |  |
| Vascular invasion | Yes | 13 |  | 24 | 0.0588 |
|  | No | 2 |  | 19 |  |
| Lymph node metastasis | Positive | 7 |  | 17 | 0.6292 |
|  | Negtive | 8 |  | 26 |  |
| Distant metastasis | Yes | 4 |  | 10 | 0.9999 |
|  | No | 11 |  | 33 |  |

*P < .05; “NA” indicates that no data were available.

**Supplementary Figure 1: ROC curves of CA19-9 for PDAC patients and non-PDAC individuals.** Solid line indicates training cohort, dashed line indicates validation cohort.


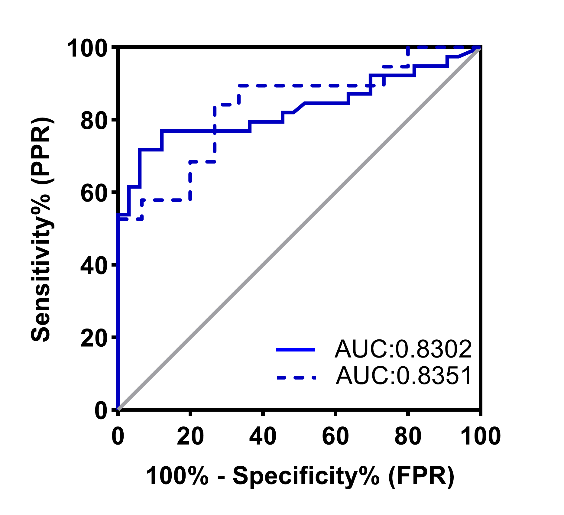


**Supplementary Figure 2: ROC curves of sEV miR-664a-3p with/without CA19-9 for PDAC patients and non-PDAC individuals.** (A-C) ROC curves of miR-664a-3p for distinguishing PDAC patients from other groups in training set (solid line) and validation set (dashed line). Non-PDAC (A), HC (B), BPD (C); (d-f) ROC curves of miR-664a-3p combined with CA19-9 for distinguishing PDAC patients from other groups in training set (solid line) and validation set (dashed line). Non-PDAC (D), HC (E), BPD (F). Solid line indicates training cohort, dashed line indicates validation cohort.


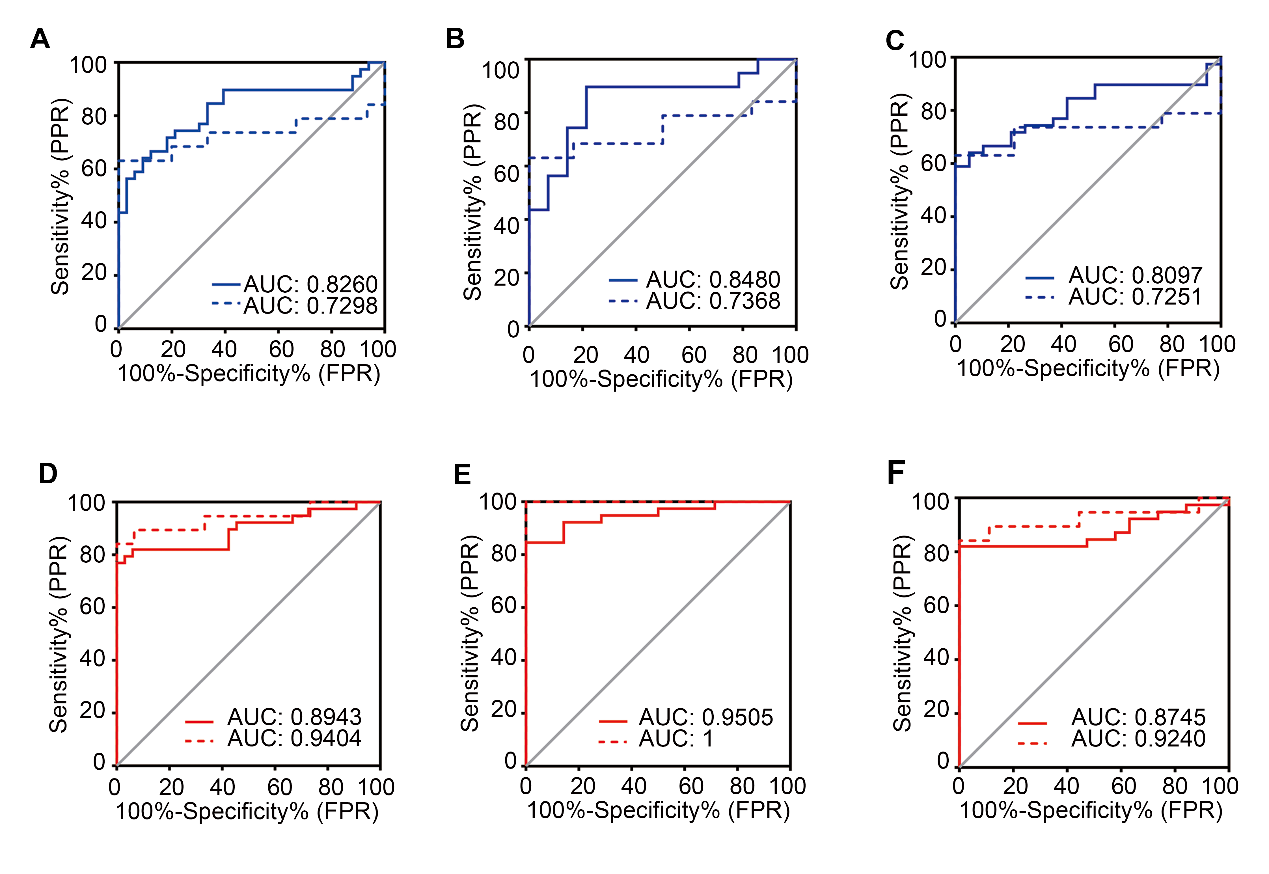


**Supplementary Figure 3: The diagnostic performance of serum miR-664a-3p in PDAC.** (A) Serum miR-664a-3p expression in 100 PDAC patients and 150 healthy controls in GSE59856. (B)ROC curve analysis of miR-664a-3p in 100 PDAC patients and 150 healthy controls in GSE59856. (C) Serum miR-664a-3p expression in 88 PDAC patients and 19 healthy controls in GSE85589. (D)ROC curve analysis of miR-664a-3p in 88 PDAC patients and 19 healthy controls in GSE85589.


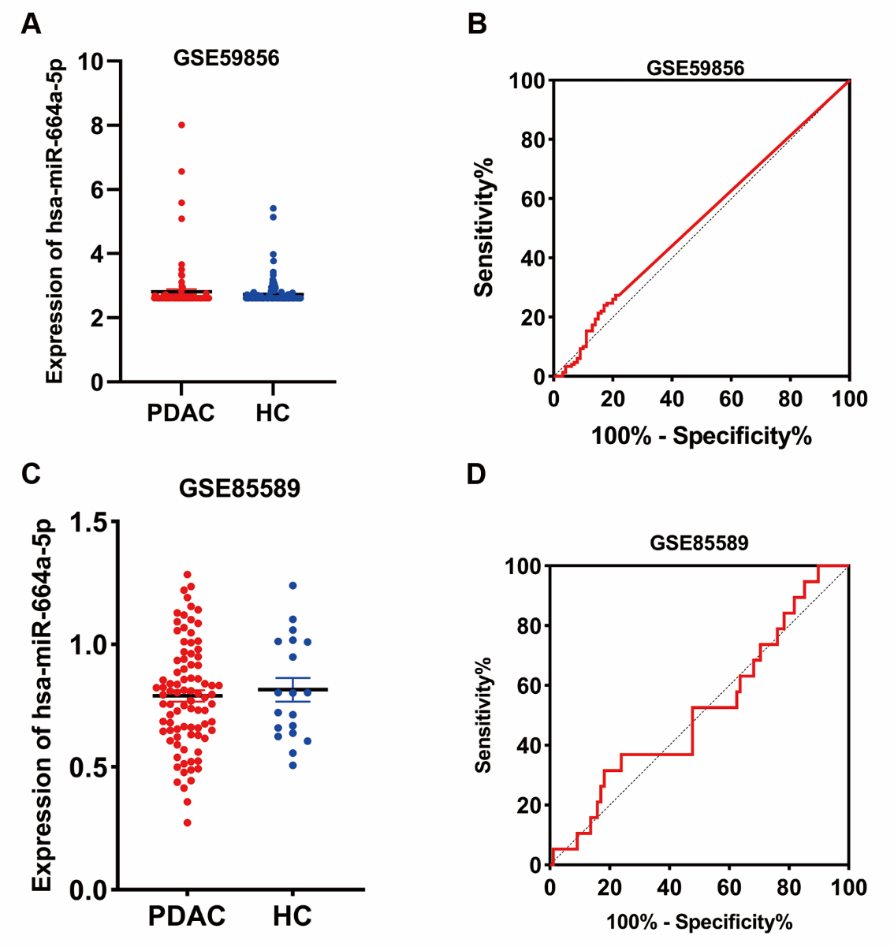


**Supplementary Figure 4: The diagnostic and prognostic performance of tissue miR-664a-3p in PDAC.** (A) Tissue miR-664a-3p expression in 178 PDAC patients and 4 healthy controls in TCGA database. (B) ROC curve analysis of tissue miR-664a-3p in 178 PDAC patients and 4 healthy controls in TCGA database. (C) K–M curve analysis of survival differences between miR-664a-3p high and low expression groups. (D) Relative expression level of miR-664a-3p in 7 patients in their cancer tissues and para-cancer tissues; (E) Paired T test was performed to analyze different expression of miR-664a-3p between cancer tissues and paracancerous tissues in 7 paired tissues; (F) Paired T test was performed to analyze different expression of miR-664a-3p between cancer tissues and para-cancerous tissues in 10 PDAC patients using GSE119794 database.


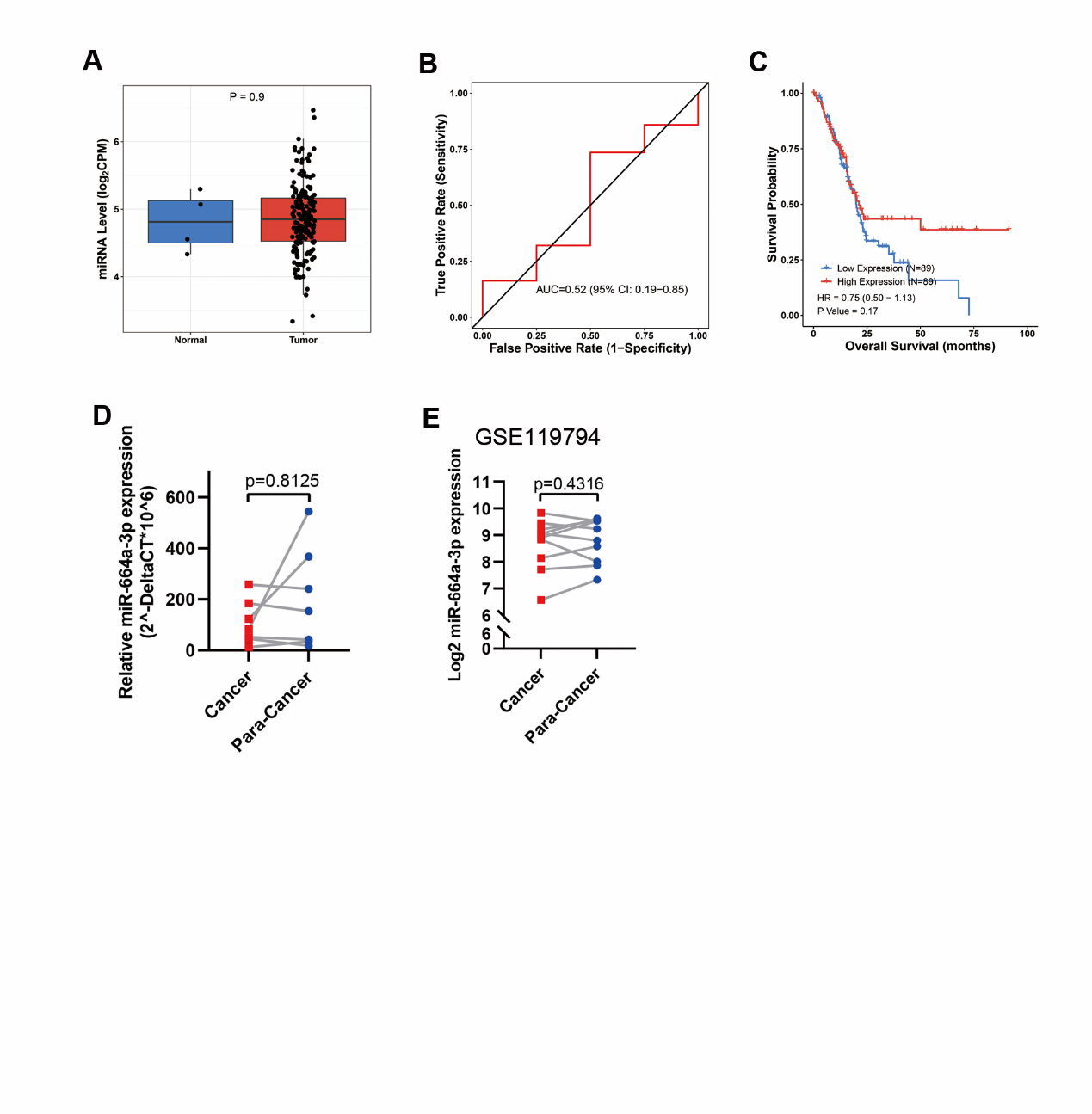


**Supplementary Figure 5: MiR-664a-3p was positively correlated with VEGFA in PDAC stroma.** A correlation analysis on the levels of miR-664a-3p in situ hybridization and IF staining for VEGFA scored from 0 to 3 in 20 different views of the PDAC stroma from 4 PDAC patients.

**
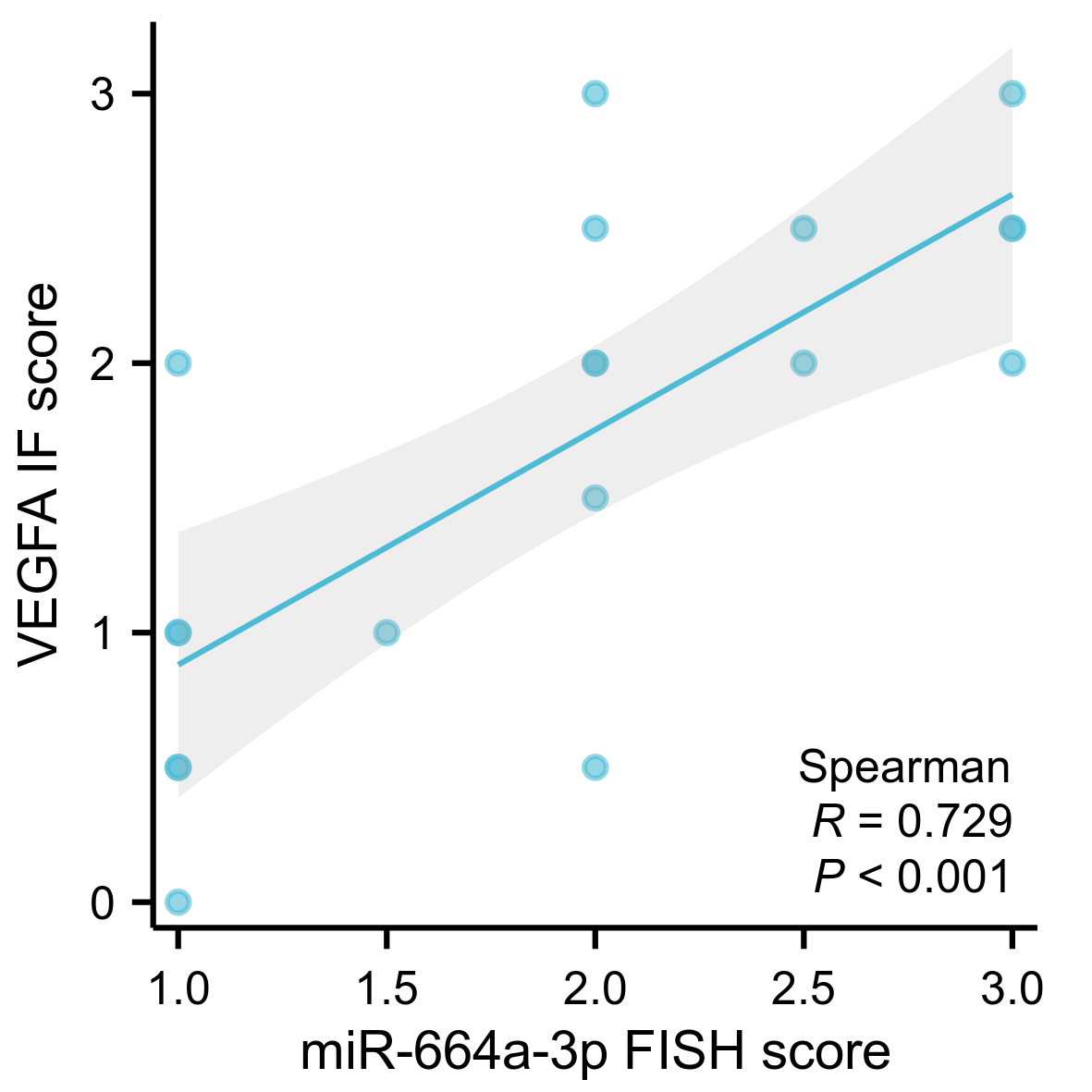
**

**Supplementary Figure 6: MiR-664a-3p have no obvious influence on the proliferation of the PDAC cell lines.** CCK-8 method was used to detect PDAC cell proliferation after transfection of miR-664a-3p mimics. (A) ASPC-1; (b) BxPC-3; (C) CFPAC-1; (D) MiaPaCa-2; (E) Panc-1.


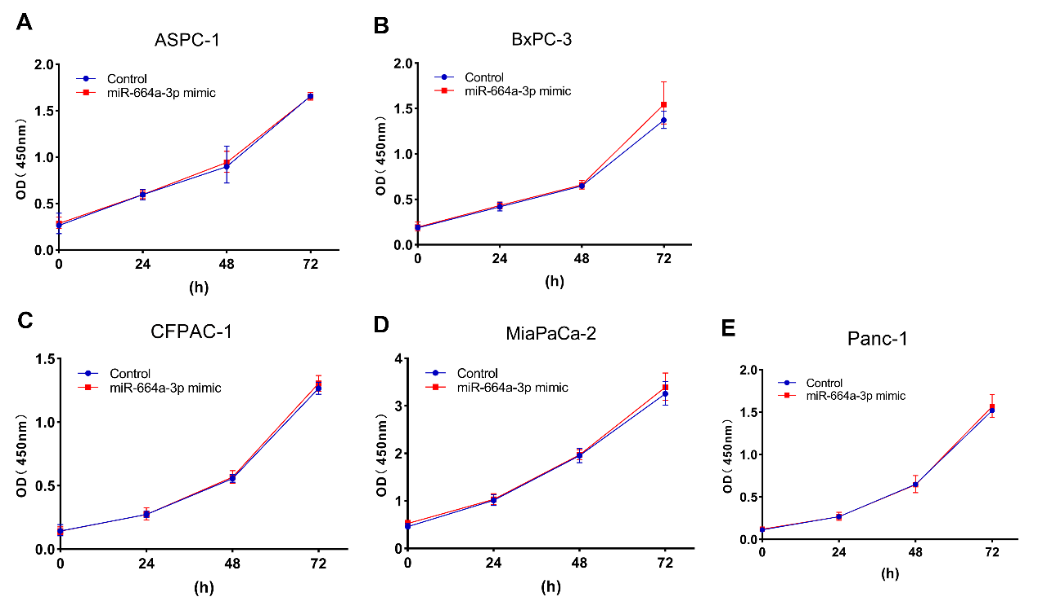

Supplement: Supplementary file 1 [file mmc1.docx]
